# Supplementary material for: Programming DNA origami patterning with non-canonical DNA-based metallization reactions
Source: Nat Commun. 2019 Dec 6;10:5597. doi: 10.1038/s41467-019-13507-5 (PMC6897912; doi:10.1038/s41467-019-13507-5)
Supplement: Supplementary file 1 — Supplementary information [file 41467_2019_13507_MOESM1_ESM.pdf]

Supplementary Information for

Programming DNA origami patterning with non-canonical DNA-based metallization reactions

Sisi Jia[1,2†], Jianbang Wang[2†], Mo Xie[2], Jixue Sun[3], Huajie Liu[4*], Yinan Zhang[2], Jie Chao[5], Jiang Li[2,6], Lihua Wang[2,7], Jianping Lin[3], Kurt V. Gothelf[8], Chunhai Fan[1*]

[1]School of Chemistry and Chemical Engineering, and Institute of Molecular Medicine, Renji Hospital, School of Medicine, Shanghai Jiao Tong University, Shanghai 200240, China

[2]Division of Physical Biology, CAS Key Laboratory of Interfacial Physics and Technology, Shanghai Synchrotron Radiation Facility, Shanghai Institute of Applied Physics, Chinese Academy of Sciences, Shanghai 201800, China.

[3]State Key Laboratory of Medicinal Chemical Biology and College of Pharmacy, Nankai University, Tianjin 300071, China.

[4]School of Chemical Science and Engineering, Key Laboratory of Advanced Civil Engineering Materials of Ministry of Education, Tongji University, Shanghai 200092, China

[5]Key Laboratory for Organic Electronics & Information Displays (KLOEID), Institute of Advanced Materials (IAM) and School of Materials Science and Engineering, Nanjing University of Posts & Telecommunications, Nanjing 210046, China.

[6]Zhangjiang Laboratory, Shanghai Advanced Research Institute, Chinese Academy of Sciences, Shanghai 201210, China

[7]Shanghai Key Laboratory of Green Chemistry and Chemical Processes, School of Chemistry and Molecular Engineering, East China Normal University, 500 Dongchuan Road, Shanghai, 200241, China

[8]Center for DNA Nanotechnology (CDNA) at the Interdisciplinary Nanoscience Center (iNANO) and the Department of Chemistry, Aarhus University, Aarhus 8000, Denmark.

[†]These authors contributed equally to this work.
*e-mail: liuhuajie@tongji.edu.cn; fanchunhai@sjtu.edu.cn

This file includes:

Supplementary Figures 1 to 33
Supplementary Notes: DNA sequences
Supplementary References

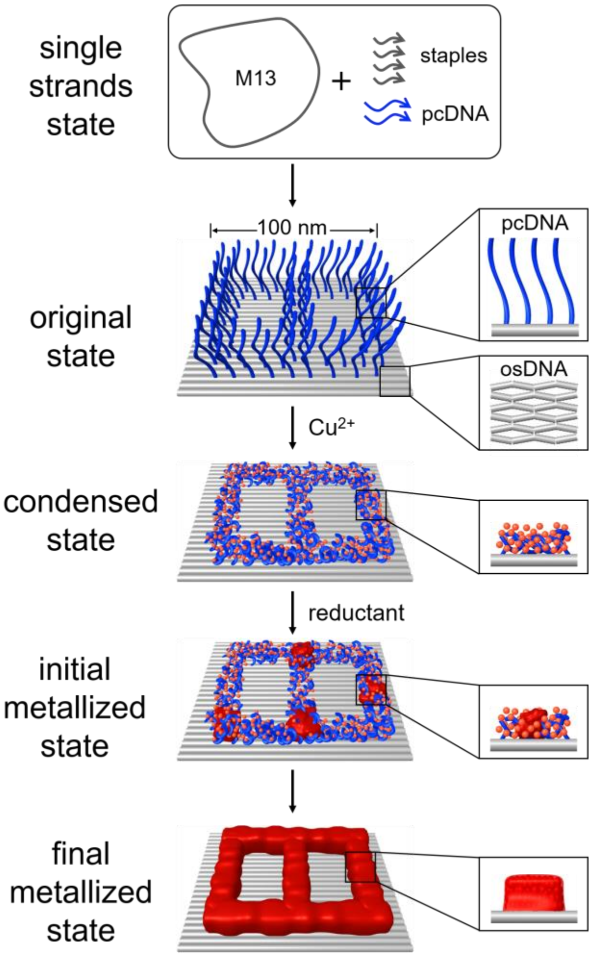

Supplementary Figure 1. Schematic illustration of Cu plating on DNA origami to form the *digit 8*.

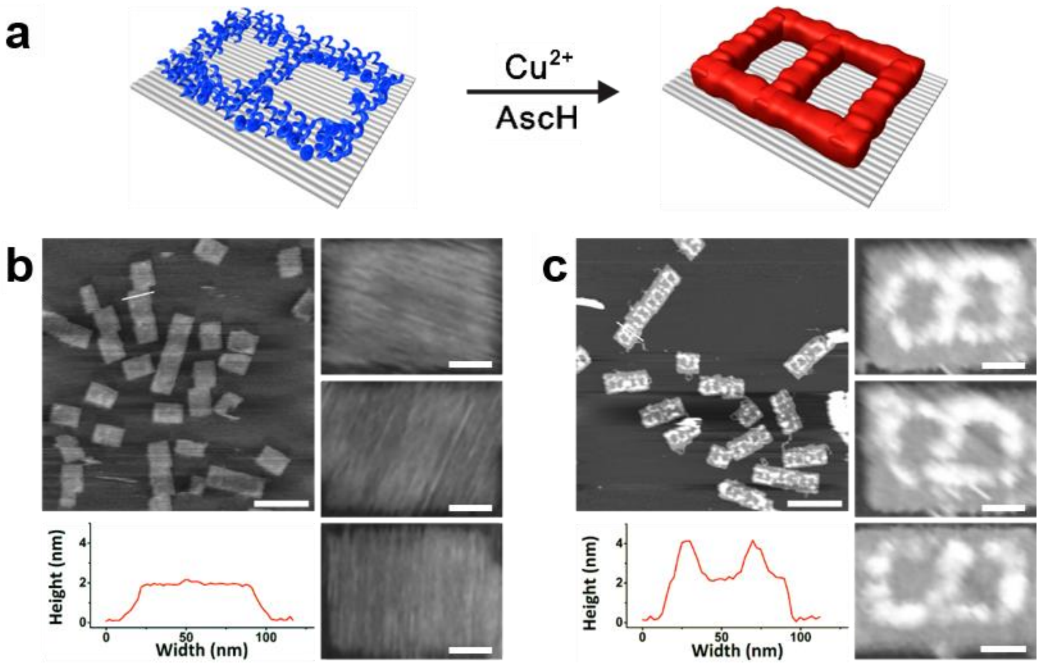

Supplementary Figure 2. Cu plating on DNA origami of *digit 8*. **a**, Schematic illustration of the formation of *digit 8* on DNA origami. **b**, AFM image showing the DNA template. **c**, AFM image showing the metal nanocircuit of *digit 8* pattern on DNA origami. Scale bars: 200 nm (large area images) and 25 nm (higher magnification images).

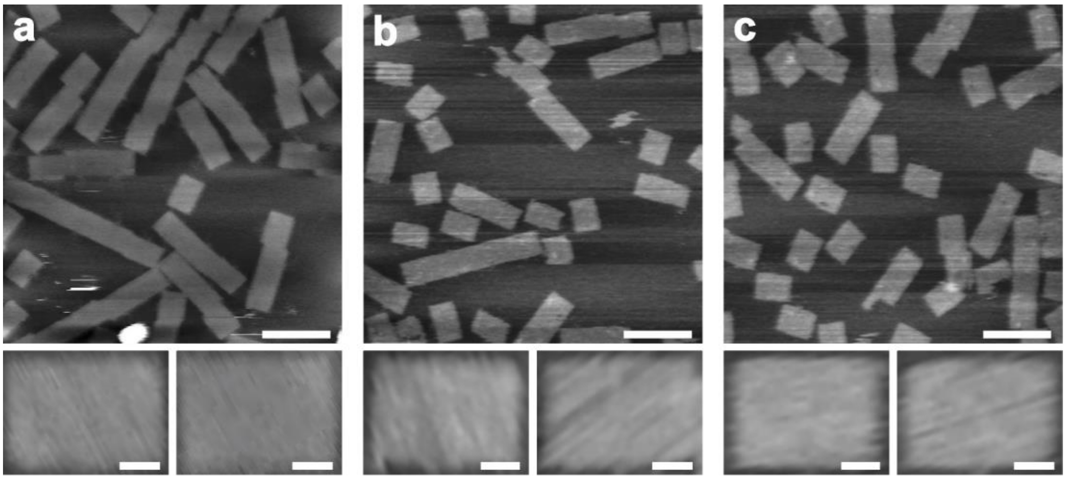

Supplementary Figure 3. Control AFM measurements of Cu plating on DNA origami. a, AFM image of copper plating reaction on a flat origami with only osDNA. **b**, AFM imgae showing no metallization on pcDNA without AscH. **c**, AFM imgae showing no metallization on pcDNA without $CuCl_2$. Scale bars: 200 nm (large area images) and 25 nm (higher magnification images).

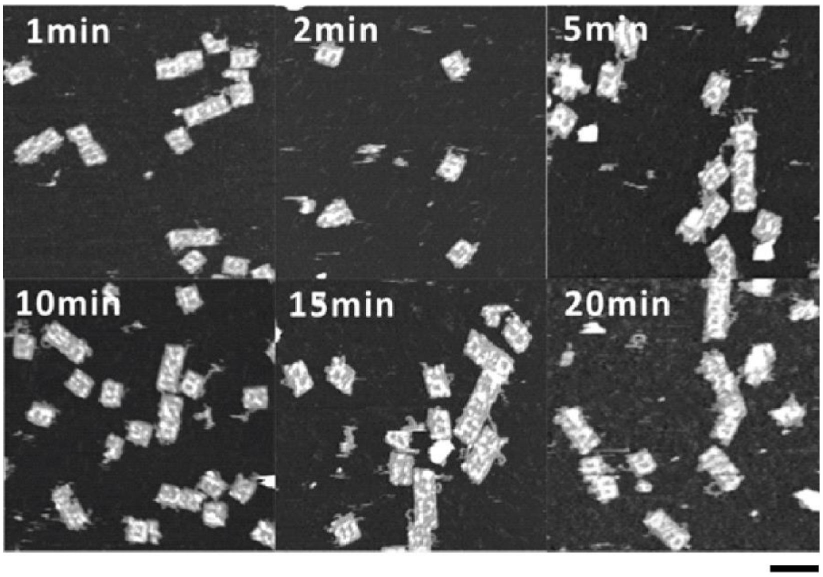

Supplementary Figure 4. Effect of reaction time on Cu metallization on DNA origami. The AFM images showed the copper plating on DNA origami of a *digit 8* pattern. Scale bar: 200 nm.

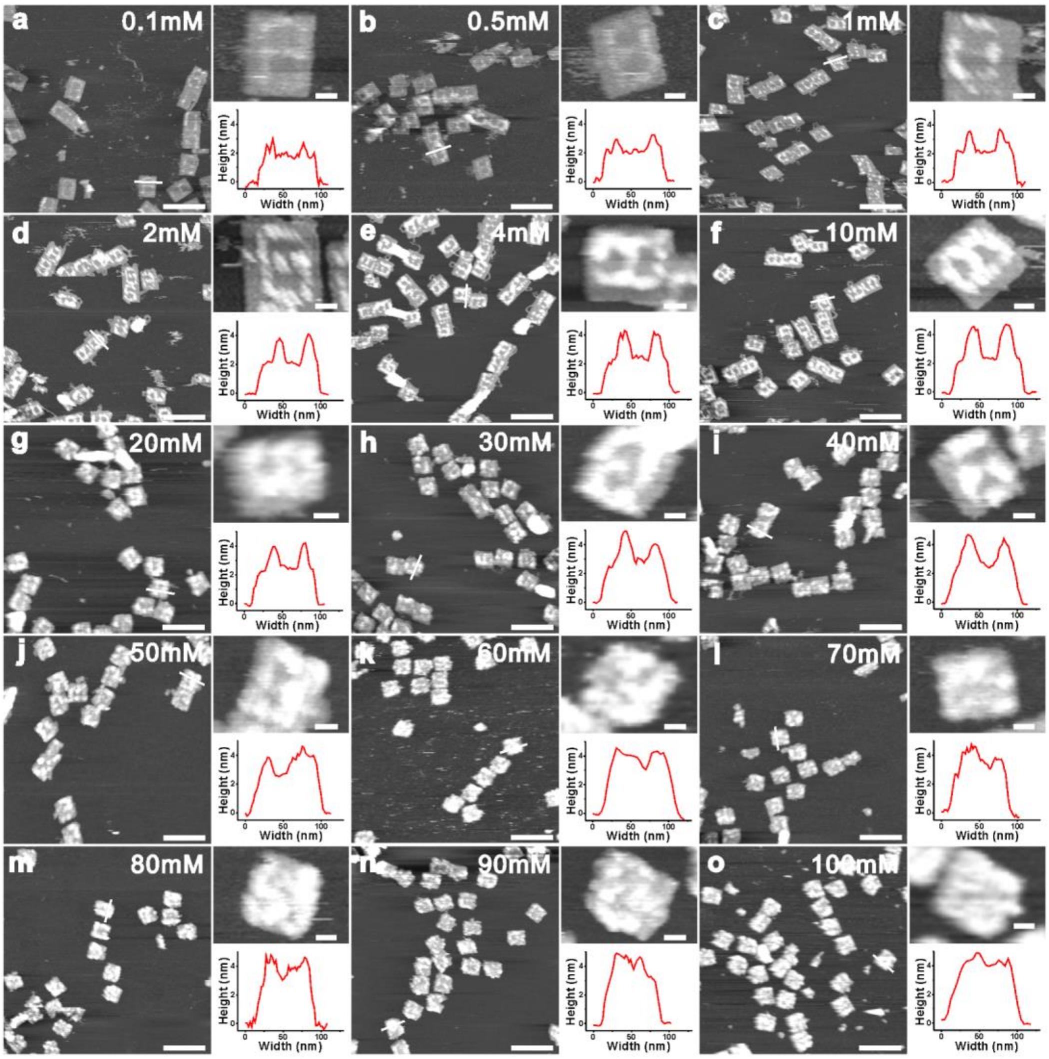

Supplementary Figure 5. Effect of Cu^{2+} concentration on Cu metallization. The concentrations for a-c were 0.1, 0.5 and 1 mM respectively. For d-f, the concentrations were 2, 4 and 10 mM respectively. The concentrations for g-o were 20, 30, 40, 50, 60, 70, 80, 90 and 100 mM respectively. Scale bars: 200 nm (large area images) and 25 nm (higher magnification images).

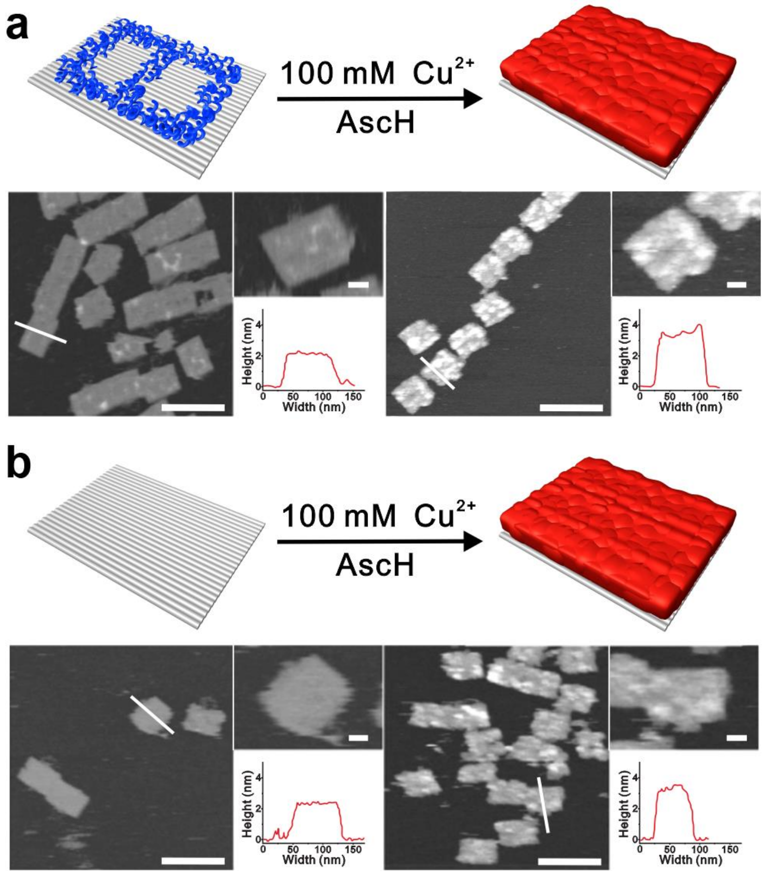

Supplementary Figure 6. Cu metallization with a high concentration of Cu^{2+} (100 mM). **a**, Copper metallization on a DNA origami of *digit 8* pattern. **b**, Metallization on a flat DNA origami without any pcDNA. Scale bars: 200 nm (large area images) and 25 nm (higher magnification images).

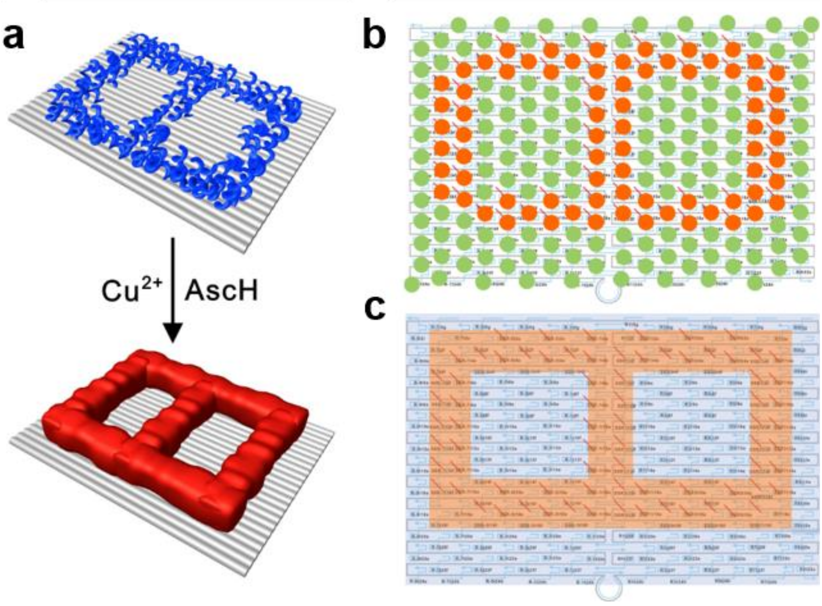

Supplementary Figure 7. Calculation of the theoretical proportion of the metalized area. **a**, Schematic illustration of the metallization on the *digit 8* pattern. **b**, Illustration of the calculation method 1: the total number of staples is 216 and the number of pcDNA is 75, therefore the proportion is 75/216 = 34.72%. **c**, Illustration of the calculation method 2: the size of this origami is 7000 nm^2 and the size of the metalized area is 2813 nm^2, therefore the proportion is 2813/7000 = 40.19%.

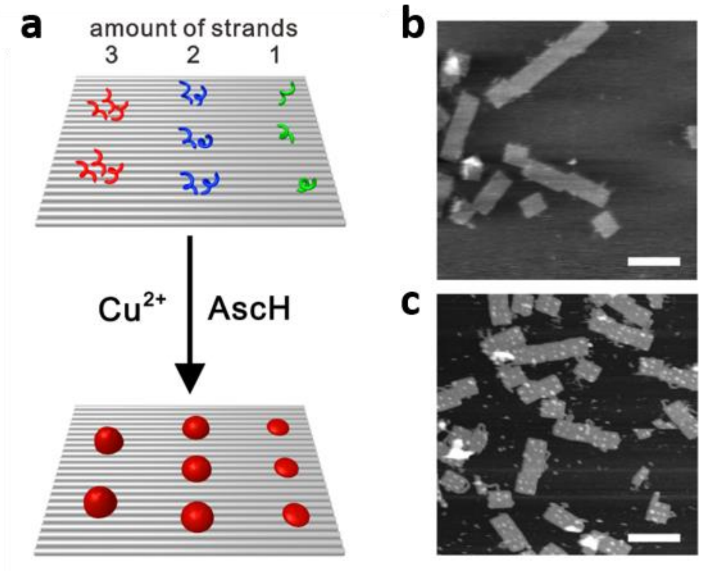

Supplementary Figure 8. Number effects of pcDNA strands per site on metallization. **a**, Schematic representation of the origami canvas with seven predefined sites containing one, two and three pcDNA strands, respectively. Each single strand contained 15 bases. **b-c**, AFM measurements for before (**b**) and after (**c**) Cu metallization. Scale bars: 200 nm.

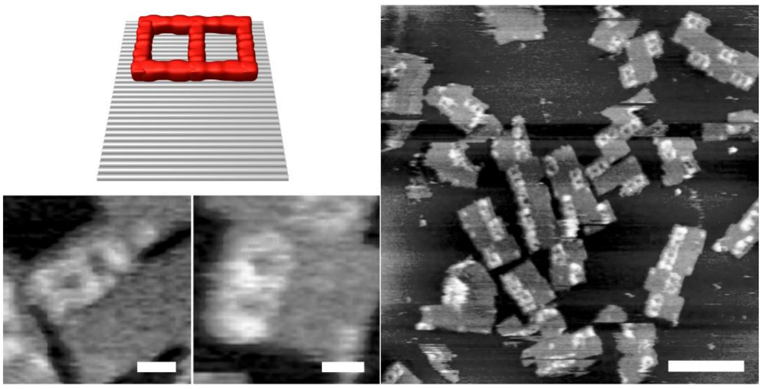

Supplementary Figure 9. Cu metallization on *miniaturized digit 8* pattern. Scale bars: 200 nm (large area image) and 25 nm (higher magnification images).

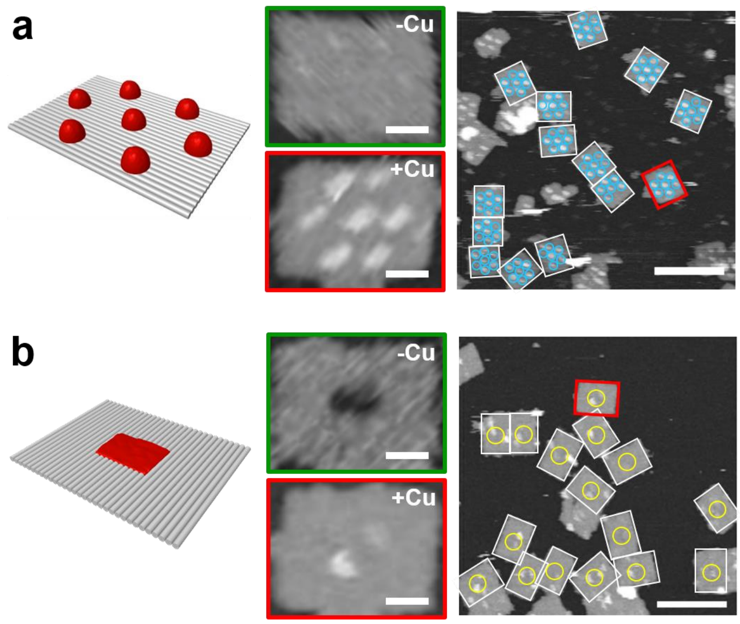

Supplementary Figure 10. Cu plating on a *seven-dot* pattern in a positive mode (a) and Cu plating on a *rectangular block* pattern in a negative mode (b). Scale bars: 200 nm (large area images) and 25 nm (higher magnification images).

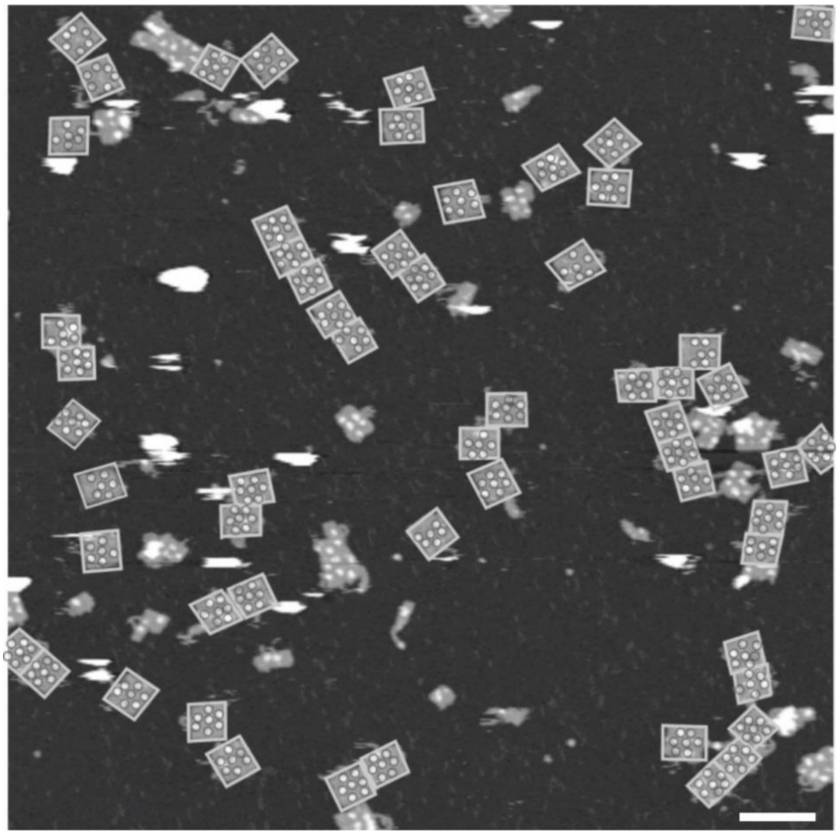

Counted origami	Theoretical number of dots	Found number of dots	Yield of Cu metallization
57	399	383	95.99%

Supplementary Figure 11. Counting metallization yield on *seven-dot* pattern. The rectangle panes and circles highlighted the DNA origami contours and the metallized dots after Cu plating process. Scale bar: 200 nm.

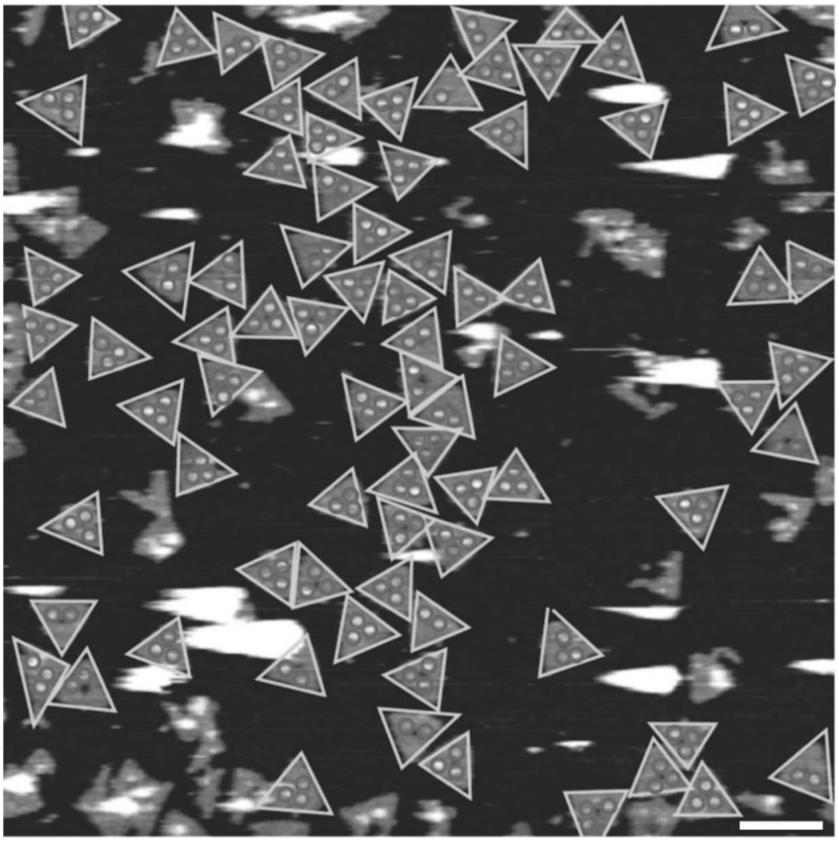

Counted origami	Theoretical number of dots	Found number of dots	Yield of Cu metallization
81	243	219	90.12%

Supplementary Figure 12. Counted yield of Cu metallization on *three-dot* pattern. The triangle panes and circles highlighted the DNA origami contours and the metallized dots after Cu plating process. Scale bar: 200 nm.

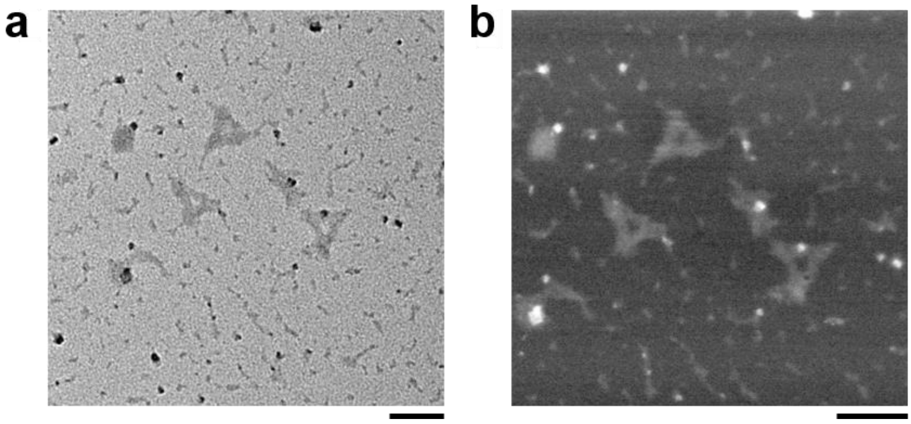

Supplementary Figure 13. TEM measurement of Cu metallization on triangular origami. a, TEM image. **b,** STEM image. Scale bars: 100 nm.

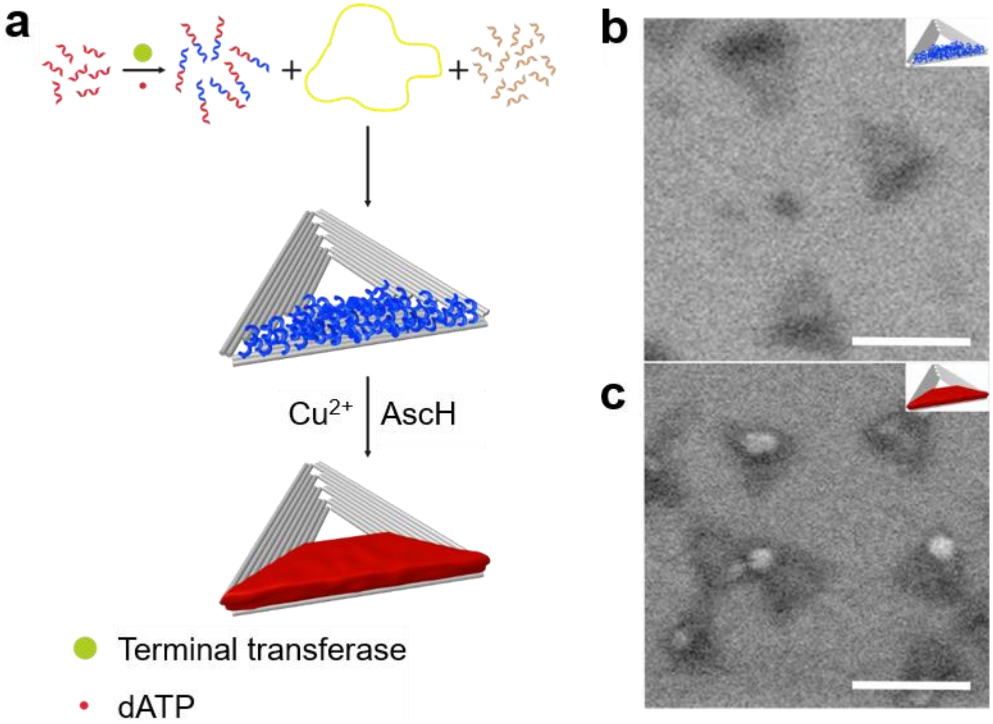

Supplementary Figure 14. SEM measurement of Cu metallization on triangular origami. **a**, Schematic illustration of the prolonging of staple strands and the plating process on DNA origami. **b-c**, SEM images showing the DNA origami and the metallization before and after plating. Scale bars: 200 nm.

a

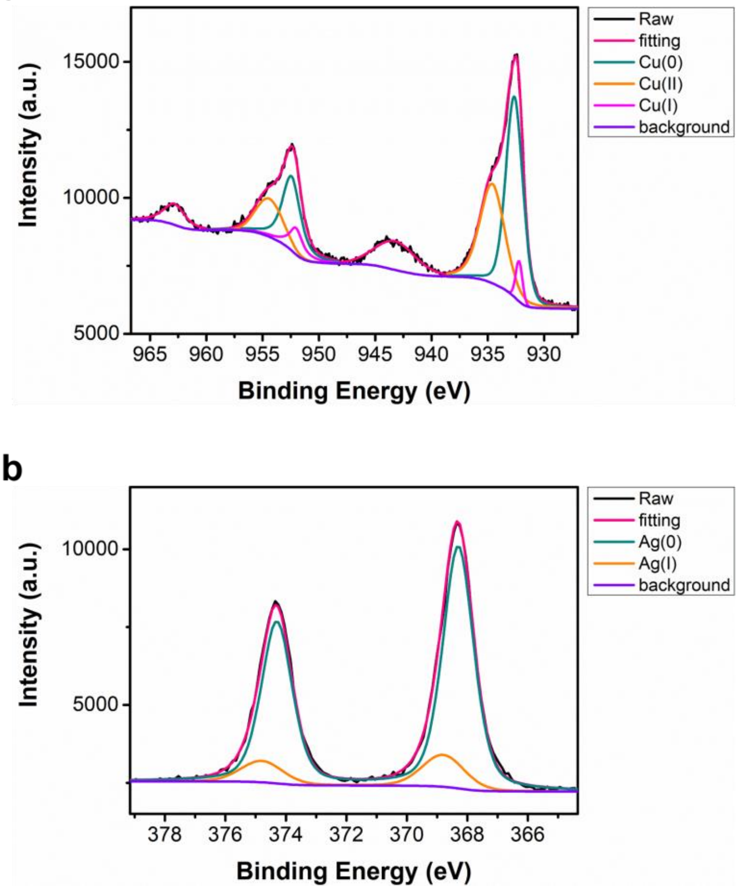

Supplementary Figure 15. X-ray Photoelectron spectra of the Cu and Ag metallization samples. a, XPS spectrum of Cu*2p* region (raw data, black curve) and the fitting model (pink curve) showing that the sample included three components: Cu(0) (dark cyan curve, 55.2%), Cu(II) (orange curve, 40.9%) and Cu(I) (magenta curve, 4.0%) species[1]. **b**, XPS spectrum of Ag*3d* region (raw data, black curve) and the fitting model (pink curve) showing that the sample included two components: Ag(0) (dark cyan curve, 86.0%) and Ag(I) (orange curve, 14.0%) species[2].

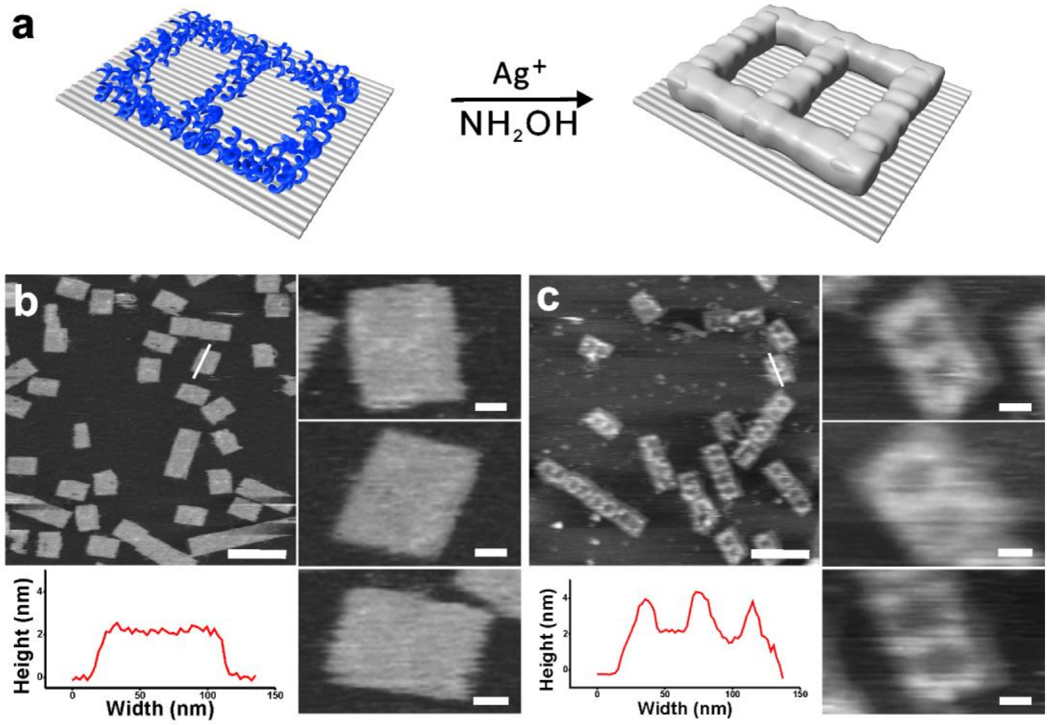

Supplementary Figure 16. Ag metallization on DNA origami of *digit 8* pattern. **a**, Schematic representation of the Ag metallization process. The mica-attached DNA origami of *digit 8* pattern was treated with the plating solution of $AgNO_3$ and NH_2OH. **b-c**, AFM images and cross-section analysis measured before (**b**) and after (**c**) Ag metallization. Scale bars: 200 nm (large area images) and 25 nm (higher magnification images).

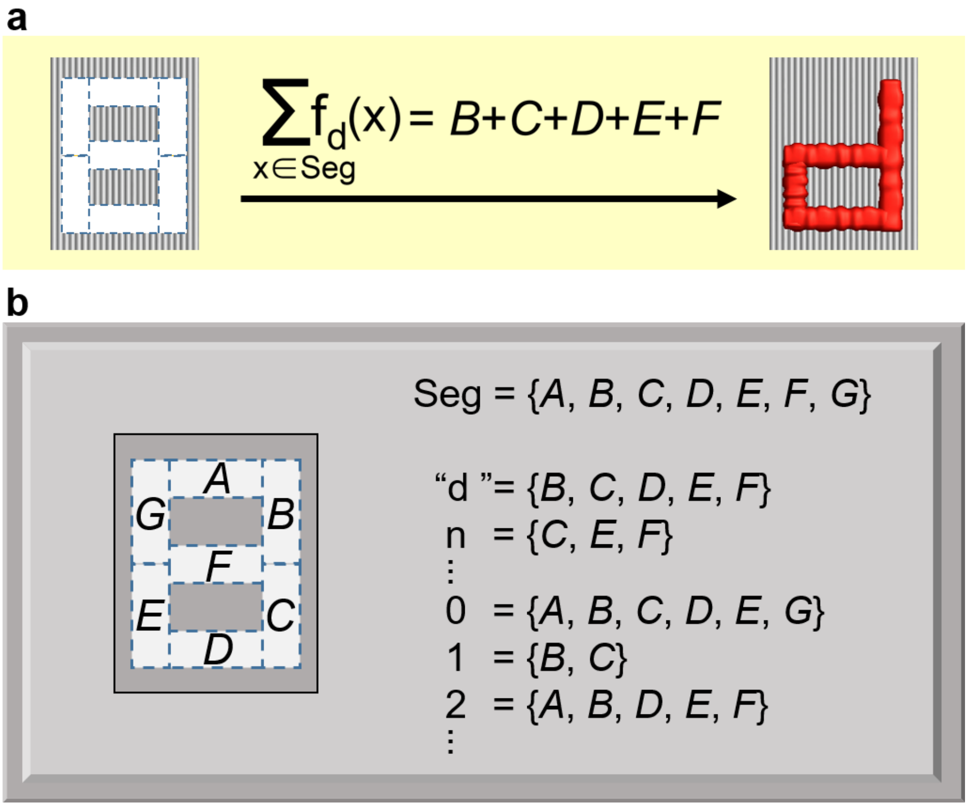

Supplementary Figure 17. Fabricating nano-PCB mimics with DCIMP. a, A *seven-segment digit 8* design was employed to fabricate nano-PCB mimics on a rectangular origami. To design an *alphabet d* pattern, we placed pcDNA on selected segments *B*, *C*, *D*, *E* and *F* (*d*=[*B*, *C*, *D*, *E*, *F*] ⊂ Seg=[*A*, *B*, *C*, *D*, *E*, *F*, *G*]). **b**, To show the generality, we fabricated digits from 0 to 9 and several typical alphabets, as shown in corresponding AFM images.

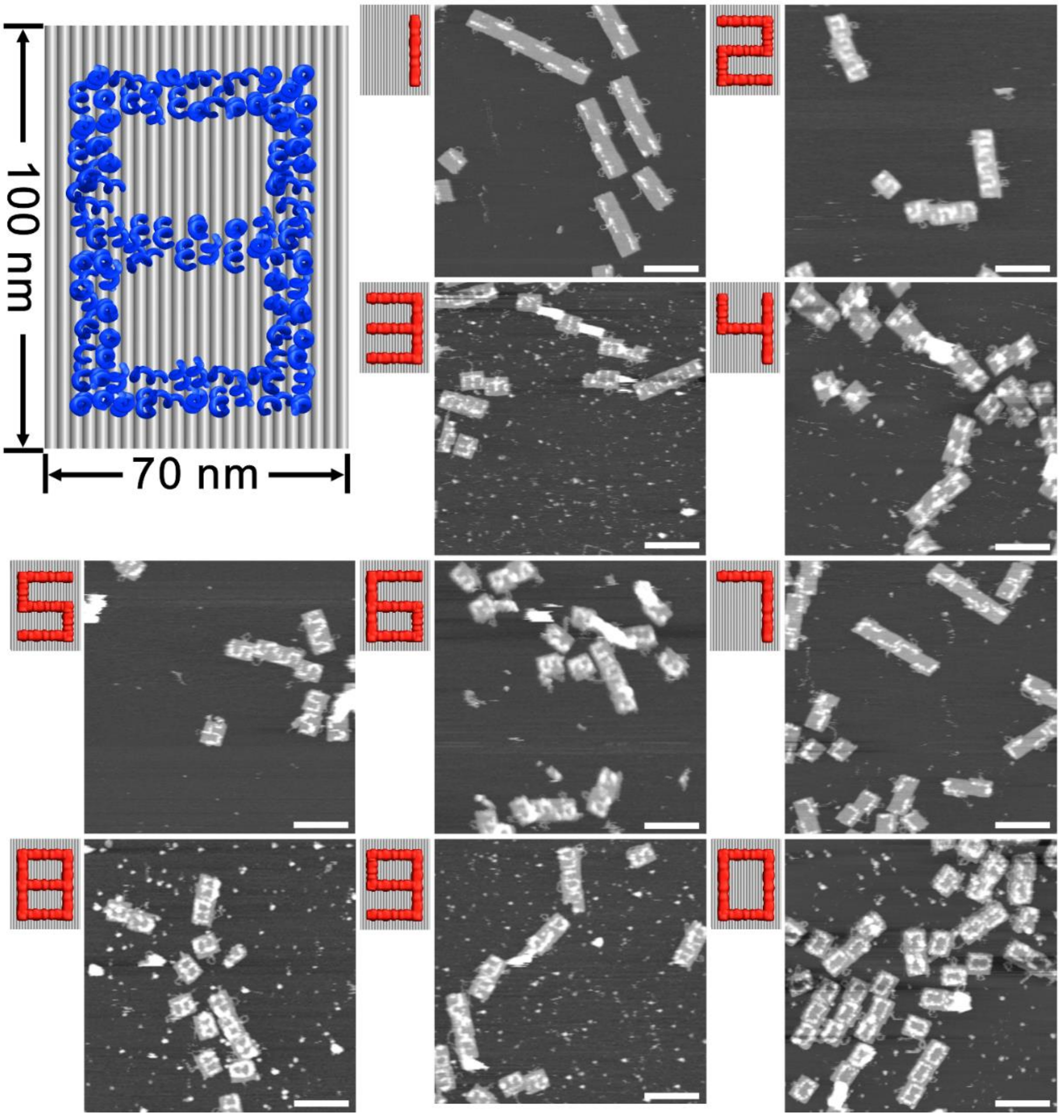

Supplementary Figure 18. Cu metallization of numerals based on nanosized *seven-segment display* pattern. AFM images showed metallized numerals from *0* to *9*. Scale bars: 200 nm.

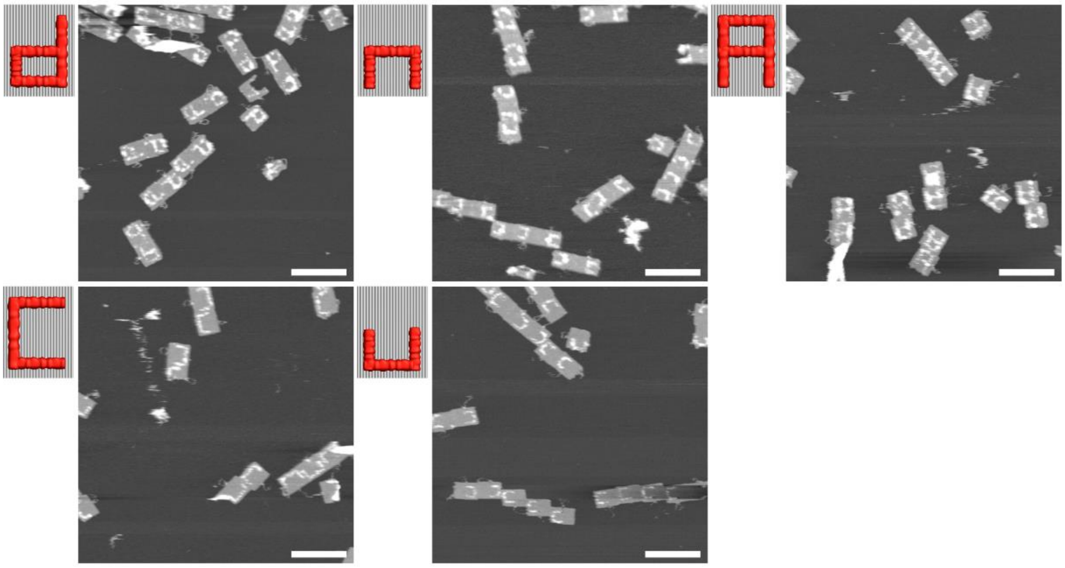

Supplementary Figure 19. Cu metallization of alphabets based on nanosized *seven-segment display* pattern. AFM images showed metalized alphabets *d*, *n*, *A* and *C*, *u*. Scale bars: 200 nm.

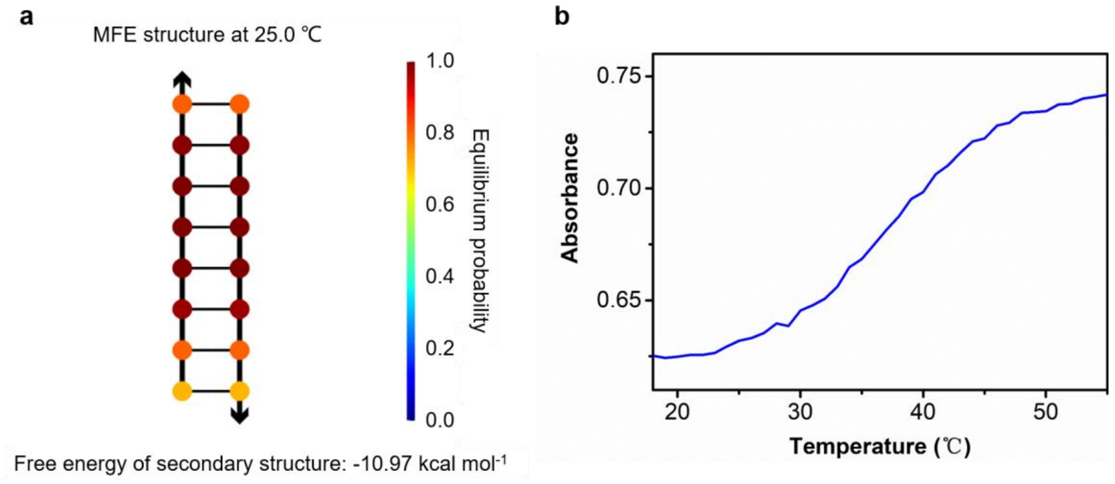

Supplementary Figure 20. Thermodynamic simulation and UV absorbance at 260 nm measurement of the short DNA sequences. a, Thermodynamic simulation of the sequences using NUPACK software with 0.05 M Na^+, 0.012 M Mg^{2+} at 25 °C. The ΔG of the double strands of the 8-base sequences was -10.97 kcal mol^{-1}. **b**, UV absorbance measurement at 260 nm of the sequences. For clear UV absorbance curve, the concentration of the strands was set at 6 μM in 1 × TA-Mg^{2+} (pH 8.0) buffer. The rate of increasing temperature was set at 1 °C min^{-1}. Tm of the sequences was 37.3 °C according the UV absorbance curve[3].

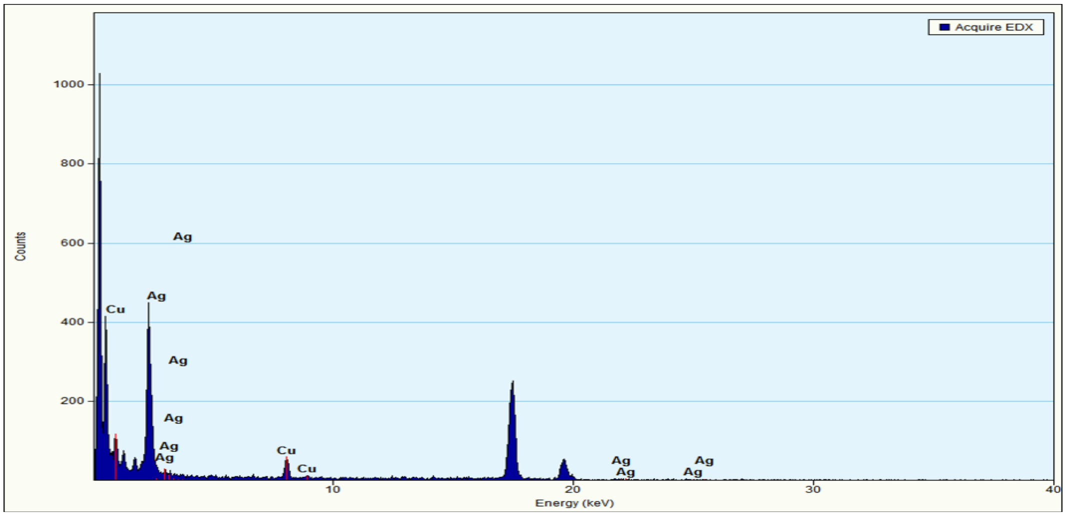

Supplementary Figure 21. EDX measurement of the metallized pattern with Ag and Cu. The elemental peaks of Cu and Ag showed that both of them were presented.

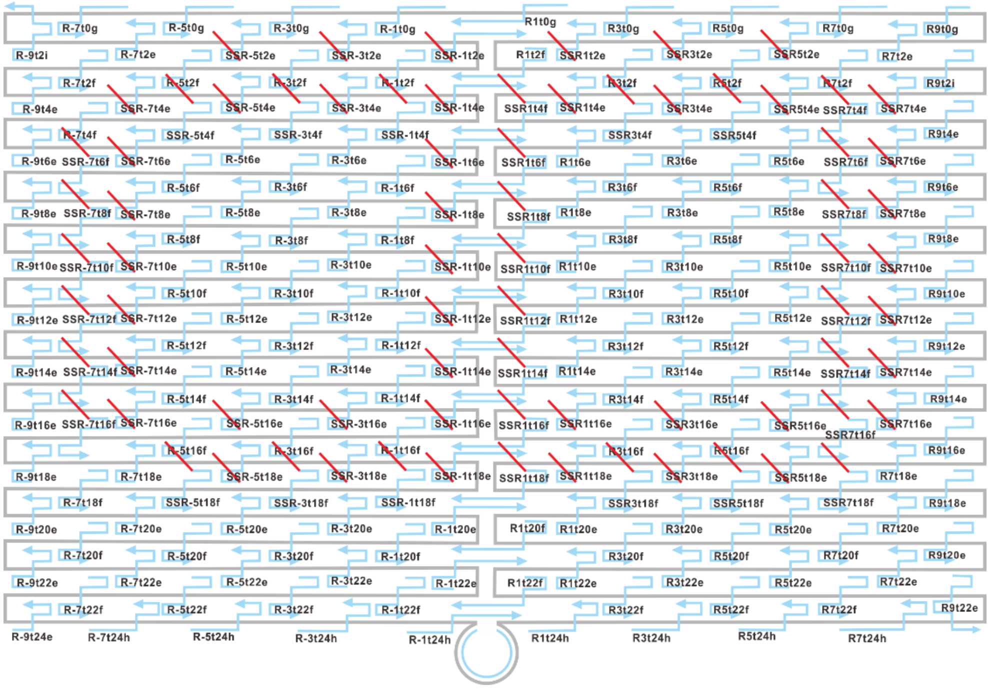

Supplementary Figure 22. Schematic representation of *seven-segment display* on rectangular DNA origami.

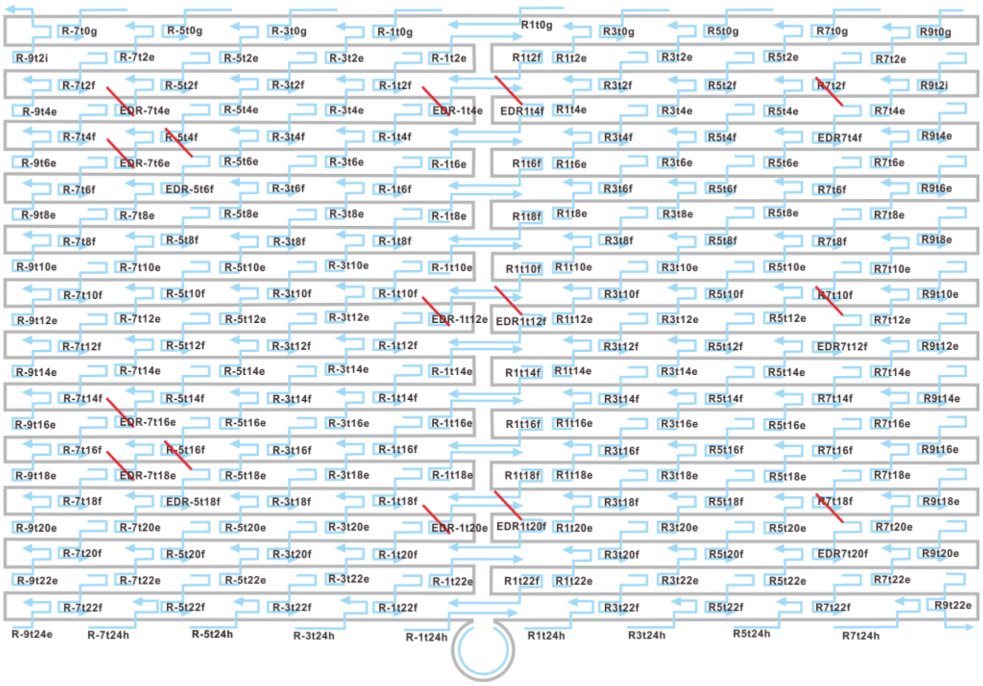

Supplementary Figure 23. Schematic representation of *eight-dot* pattern on rectangular DNA origami with varied amount of protruding ssDNA strands at each site.

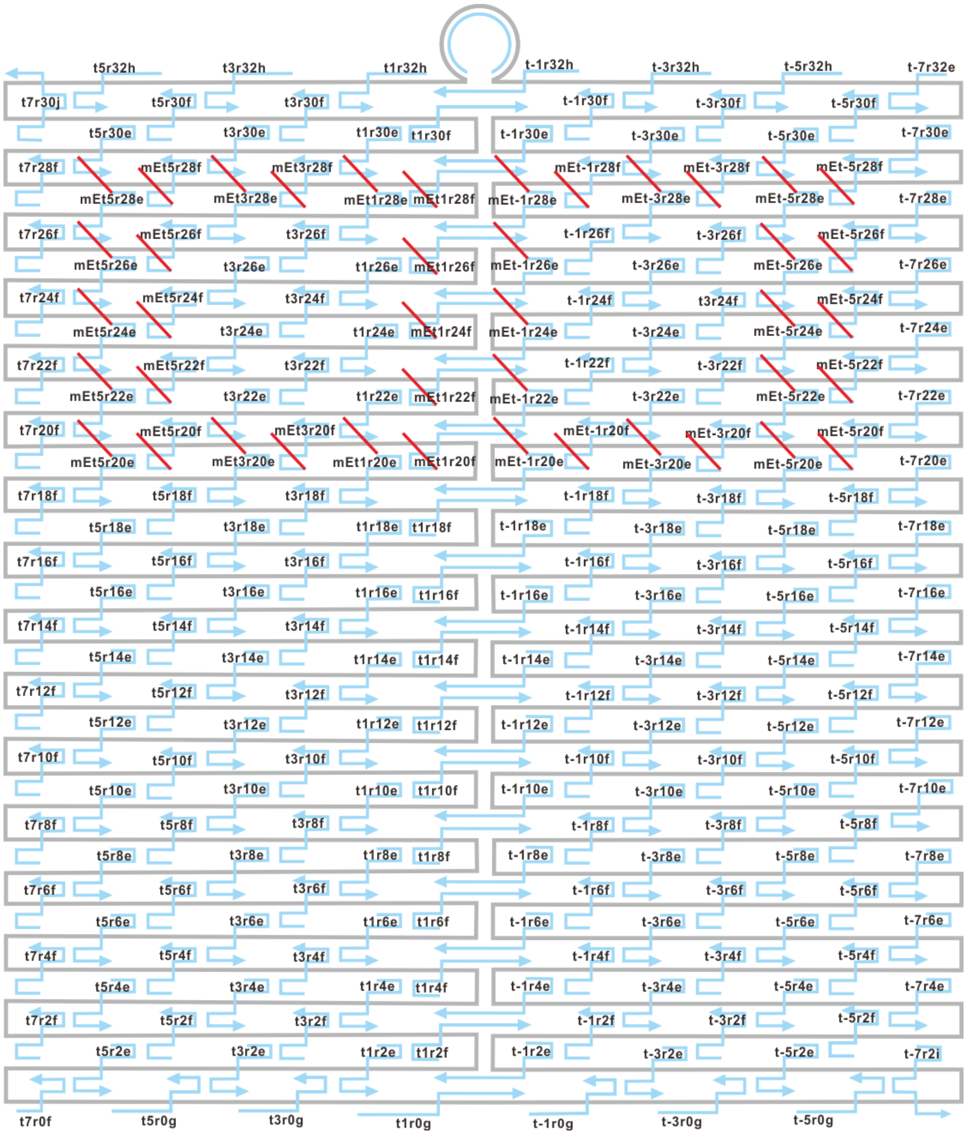

Supplementary Figure 24. Schematic representation of *miniaturized digit 8* pattern on tall rectangular DNA origami.

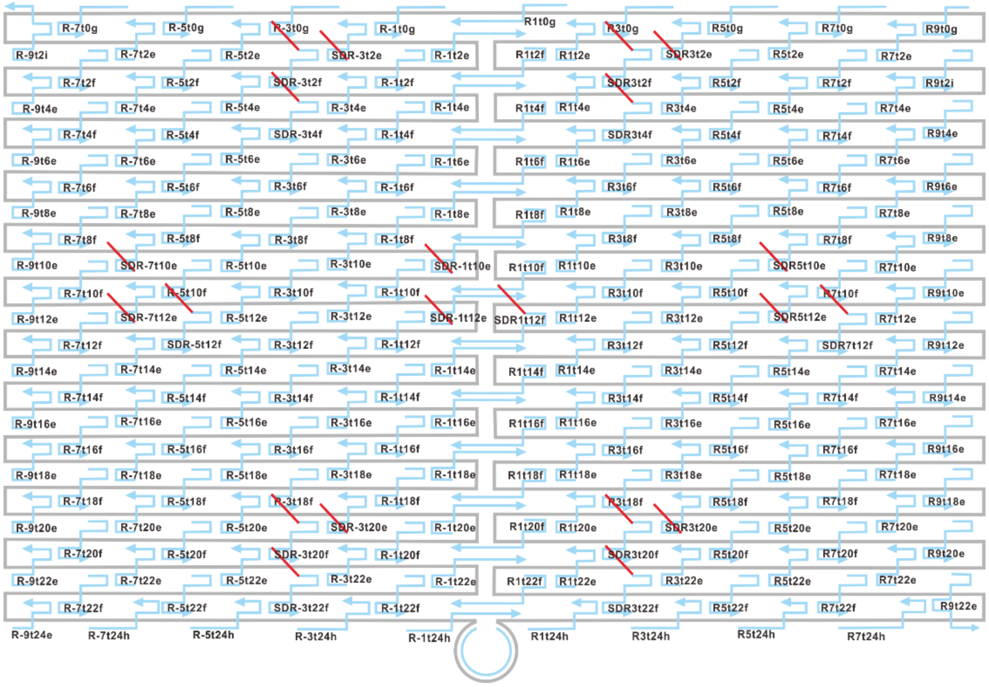

Supplementary Figure 25. Schematic representation of *seven-dot* pattern on rectangular DNA origami.

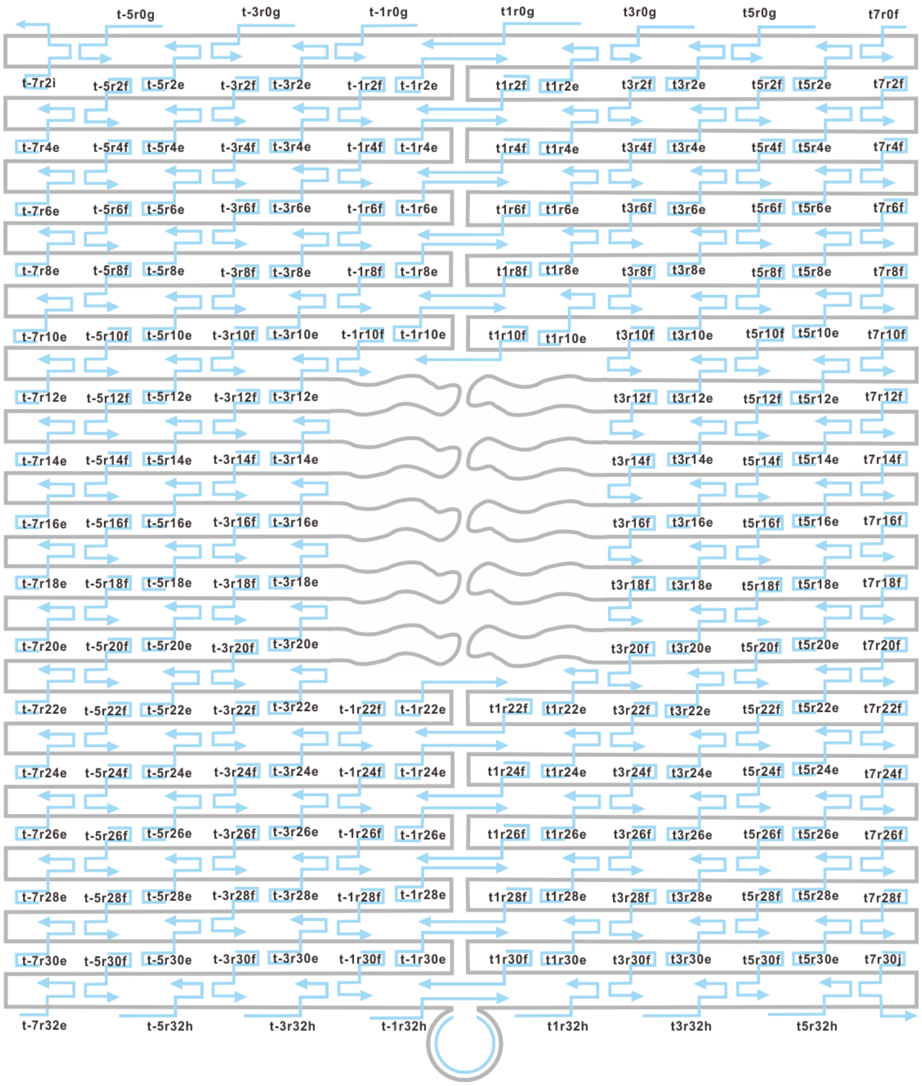

Supplementary Figure 26. Schematic representation of *rectangular block* pattern on tall rectangular DNA origami.

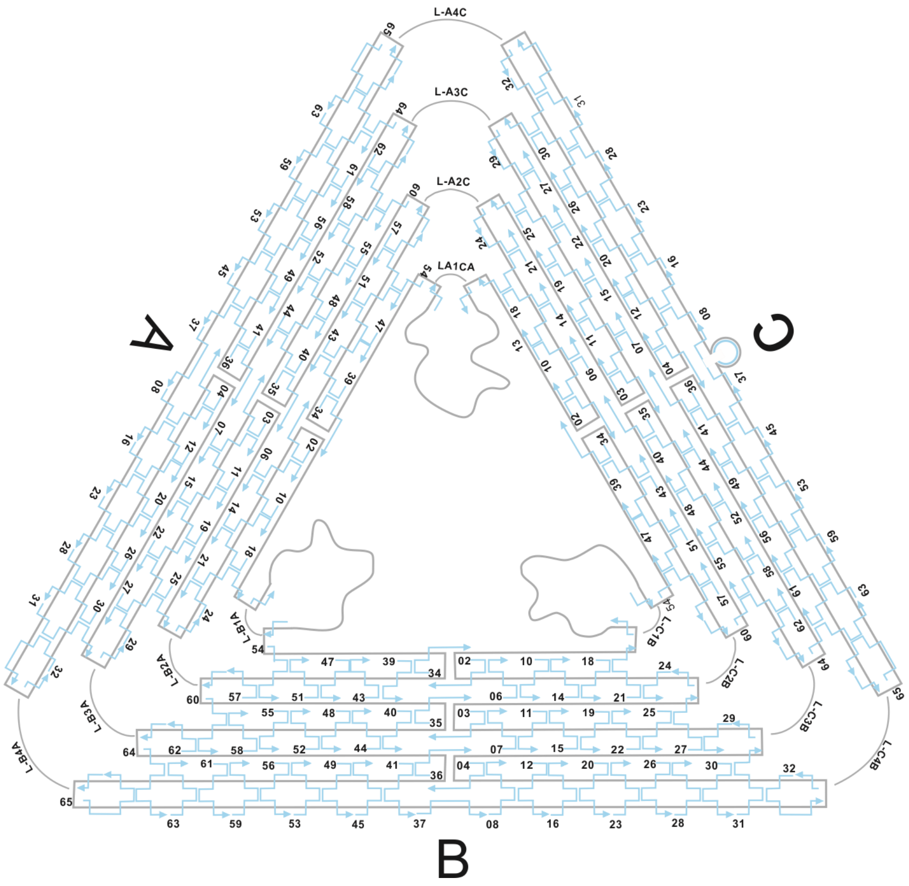

Supplementary Figure 27. Schematic representation of *three-dot* pattern on triangular DNA origami.

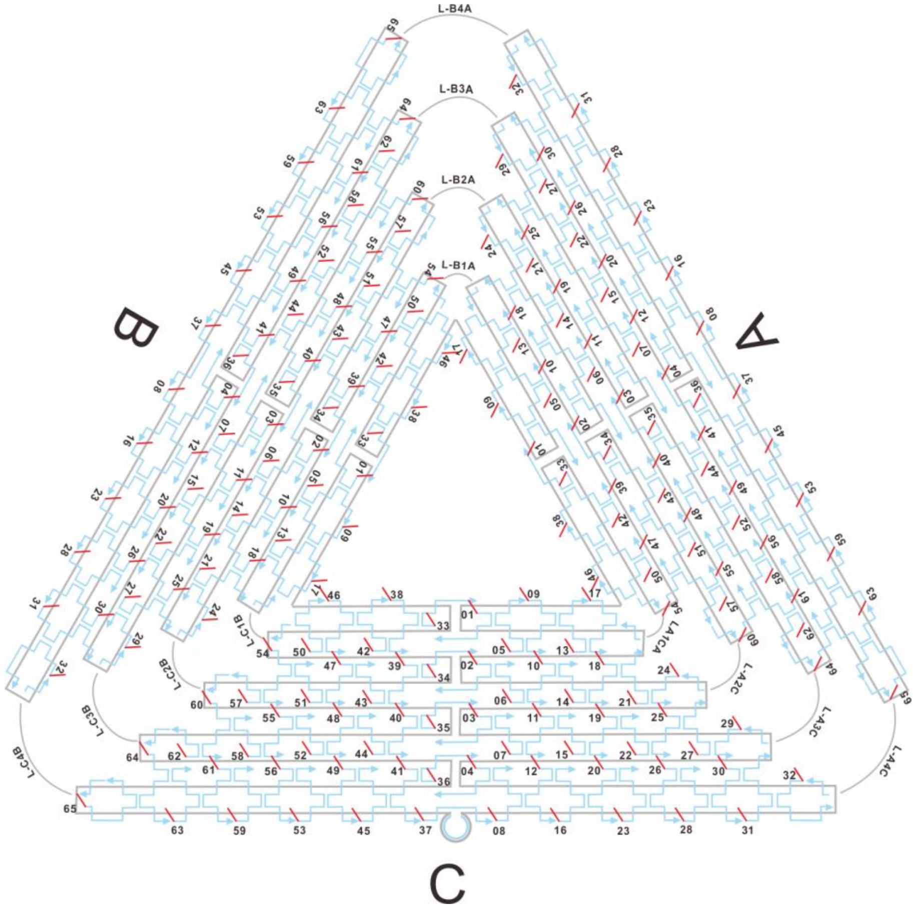

Supplementary Figure 28. Schematic representation of triangle pattern on triangular DNA origami for STEM measurements of Cu and Ag elementals.

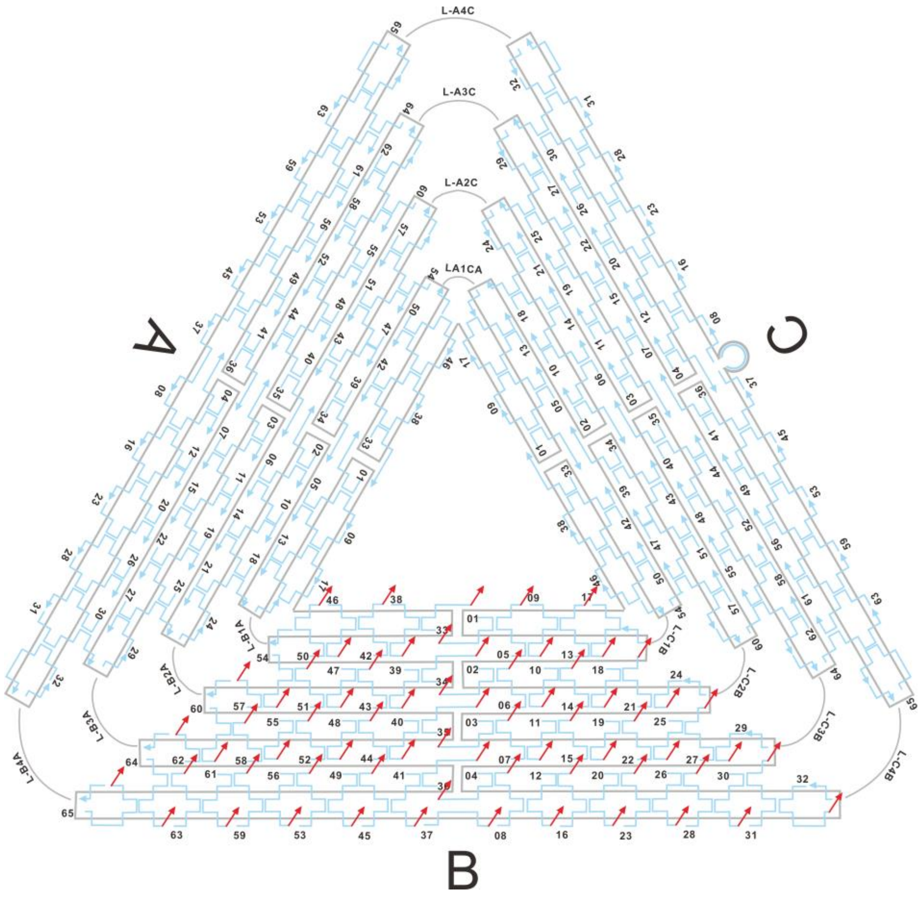

Supplementary Figure 29. Schematic representation of the TdT catalyzed elongation of poly-dA overhangs on one arm of the triangle.

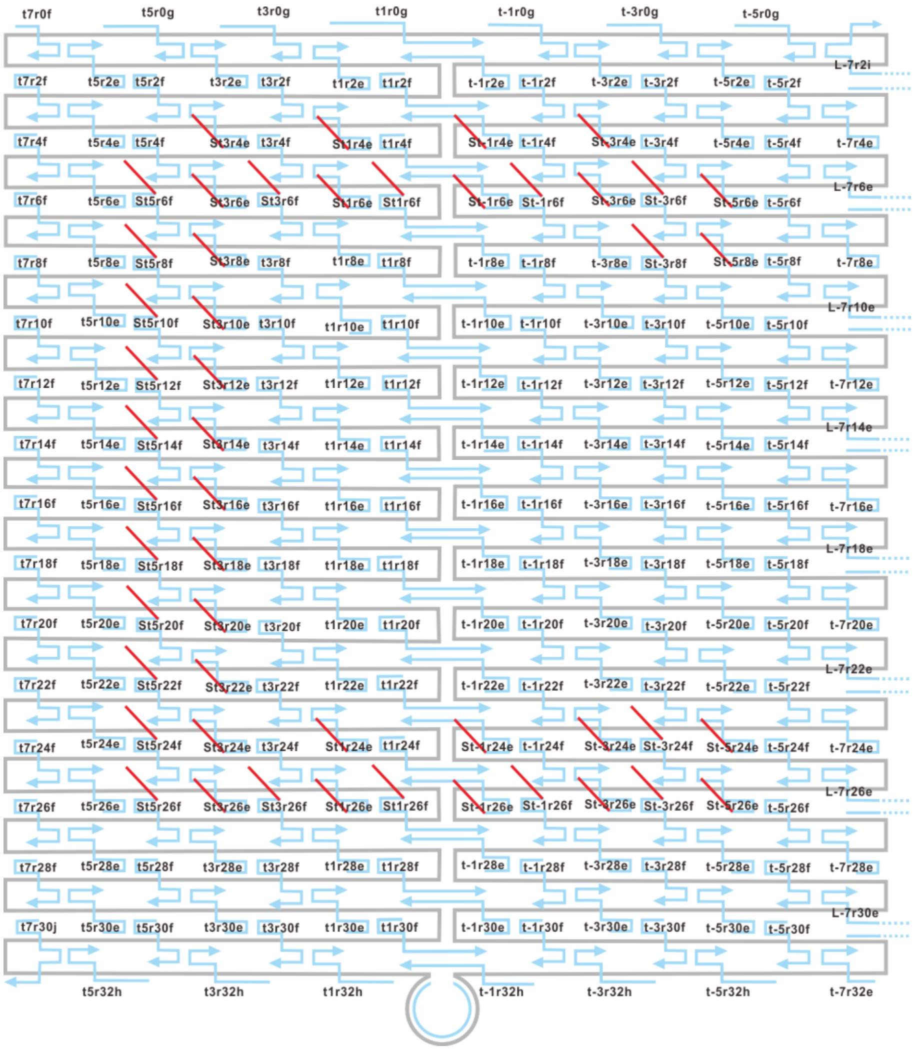

Supplementary Figure 30. Schematic representation of *C* pattern on tall rectangular DNA origami. The dashed lines on the right were used for the assembly of the origami.

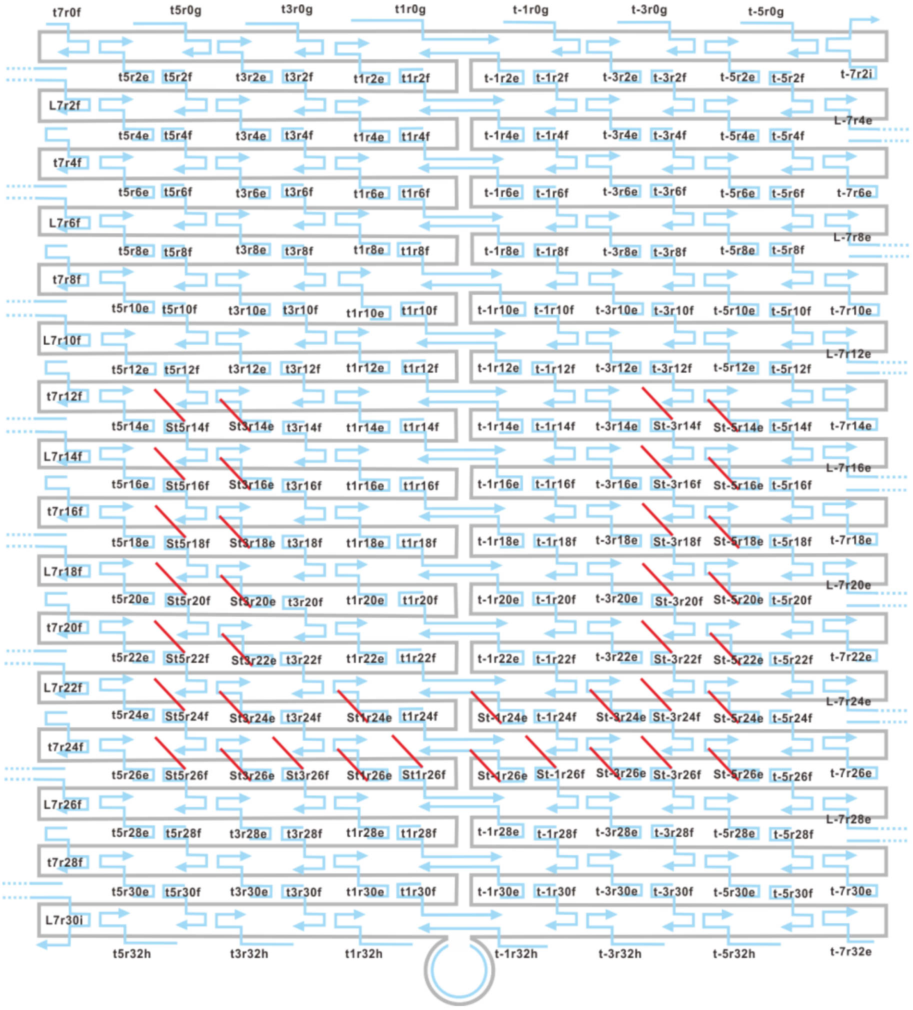

Supplementary Figure 31. Schematic representation of *u* pattern on tall rectangular DNA origami. The dashed lines were used for the assembly of the origami.

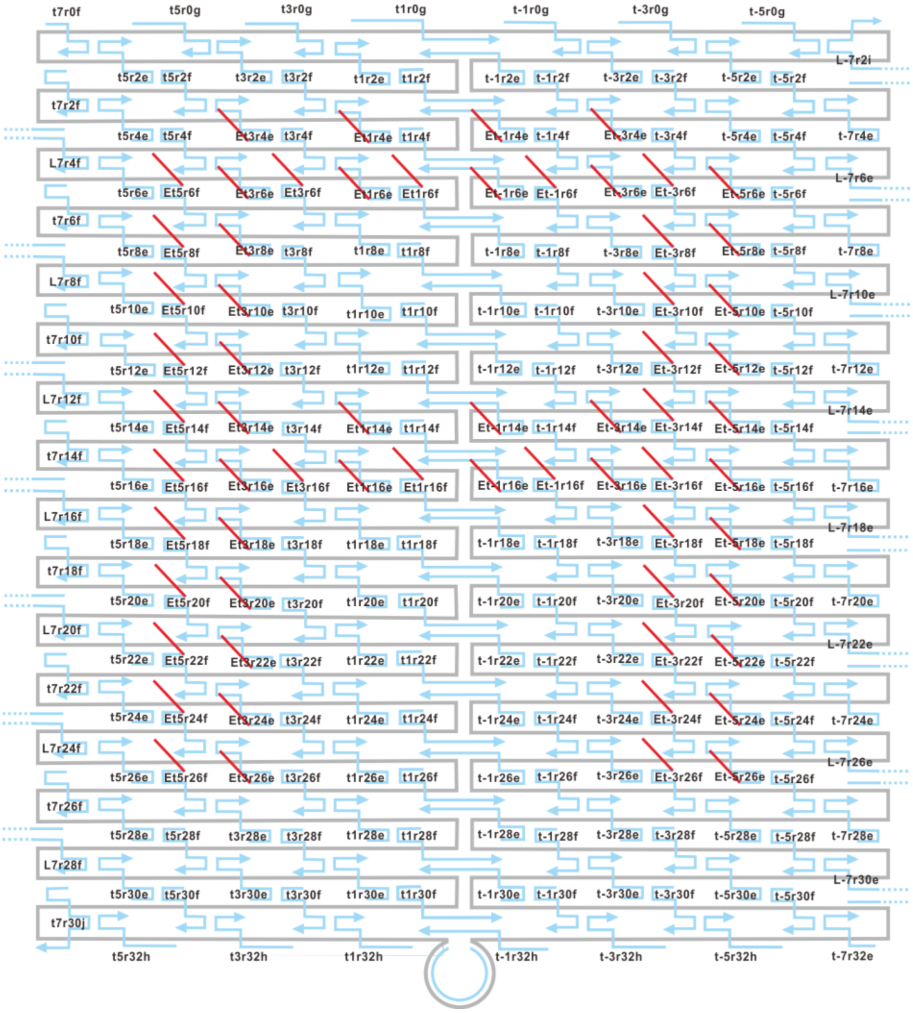

Supplementary Figure 32. Schematic representation of *A* pattern on tall rectangular DNA origami. The dashed lines were used for the assembly of the origami.

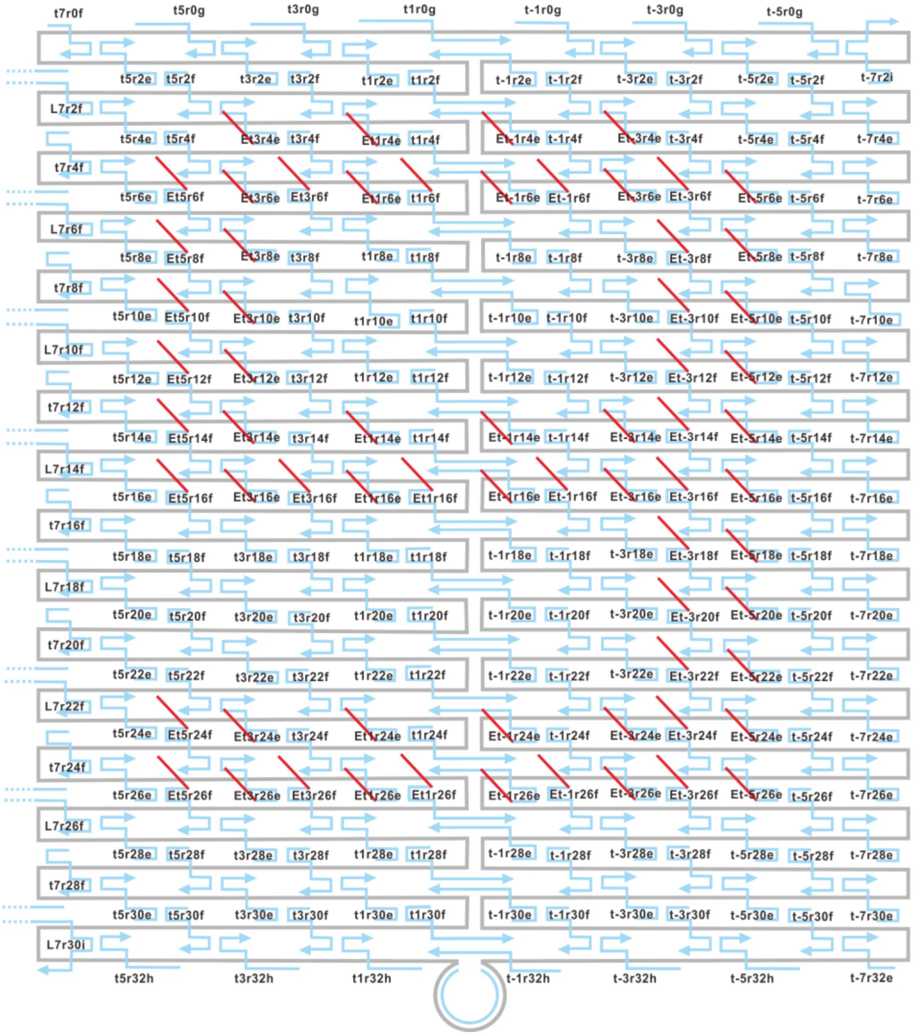

Supplementary Figure 33. Schematic representation of *g* pattern on tall rectangular DNA origami. The dashed lines were used for the assembly of the origami.

Supplementary Notes:

DNA sequences:

For *seven-segment display*:

SSR-7t6f: GACCTGACGACATAGACTTGAGAGAGCGACCTTTGAAAAGAACTGGCTCATTATTTAATAAA
SSR-7t8f: GACCTGACGACATAGACTTGAGAGAGCGACACGAACTAGCGTCCAATACTGCGGAATGCTTT
SSR-7t10f: GACCTGACGACATAGACTTGAGAGAGCGACAAACAGTTGATGGCTTAGAGCTTATTTAAATA
SSR-7t12f: GACCTGACGACATAGACTTGAGAGAGCGACTGCAACTAAGCAATAAAGCCTCAGTTATGACC
SSR-7t14f: GACCTGACGACATAGACTTGAGAGAGCGACCTGTAATATTGCCTGAGAGTCTGGAAAACTAG
SSR-7t16f: GACCTGACGACATAGACTTGAGAGAGCGACCATGTCAAGATTCTCCGTGGGAACCGTTGGTG
SSR-7t4e: GACCTGACGACATAGACTTGAGAGAGCGACGCTCCATGAGAGGCTTTGAGGACTAGGGAGTT
SSR-7t6e: GACCTGACGACATAGACTTGAGAGAGCGACCGATTTTAGAGGACAGATGAACGGCGCGACCT
SSR-7t8e: GACCTGACGACATAGACTTGAGAGAGCGACACTGGATAACGGAACAACATTATTACCTTATG
SSR-7t10e: GACCTGACGACATAGACTTGAGAGAGCGACTTTTTGCGCAGAAAACGAGAATGAATGTTTAG
SSR-7t12e: GACCTGACGACATAGACTTGAGAGAGCGACCAAAATTAAAGTACGGTGTCTGGAAGAGGTCA
SSR-7t14e: GACCTGACGACATAGACTTGAGAGAGCGACTCAGGTCACTTTTGCGGGAGAAGCAGAATTAG
SSR-7t16e: GACCTGACGACATAGACTTGAGAGAGCGACACCCGTCGTCATATGTACCCCGGTAAAGGCTA
SSR-5t4f: GACCTGACGACATAGACTTGAGAGAGCGACTTTCATGAAAATTGTGTCGAAATCTGTACAGA
SSR-5t18f: GACCTGACGACATAGACTTGAGAGAGCGACGTTTGAGGGAAAGGGGGATGTGCTAGAGGATC
SSR-5t2e: GACCTGACGACATAGACTTGAGAGAGCGACATATATTCTTTTTCACGTTGAAAATAGTTAG
SSR-5t4e: GACCTGACGACATAGACTTGAGAGAGCGACCGCCTGATGGAAGTTTCCATTAAACATAACCG
SSR-5t16e: GACCTGACGACATAGACTTGAGAGAGCGACCTTTCATCCCCAAAAACAGGAAGACCGGAGAG
SSR-5t18e: GACCTGACGACATAGACTTGAGAGAGCGACCAGCTGGCGGACGACGACAGTATCGTAGCCAG
SSR-3t4f: GACCTGACGACATAGACTTGAGAGAGCGACATACGTAAAAGTACAACGGAGATTTCATCAAG
SSR-3t18f: GACCTGACGACATAGACTTGAGAGAGCGACGAAGATCGGTGCGGGCCTCTTCGCAATCATGG
SSR-3t2e: GACCTGACGACATAGACTTGAGAGAGCGACCAATGACACTCCAAAAGGAGCCTTACAACGCC
SSR-3t4e: GACCTGACGACATAGACTTGAGAGAGCGACGCGAAACATGCCACTACGAAGGCATGCGCCGA
SSR-3t16e: GACCTGACGACATAGACTTGAGAGAGCGACAAATAATTTTAAATTGTAAACGTTGATATTCA
SSR-3t18e: GACCTGACGACATAGACTTGAGAGAGCGACGGCGATCGCACTCCAGCCAGCTTTGCCATCAA
SSR-1t2f: GACCTGACGACATAGACTTGAGAGAGCGACTCGGTTTAGCTTGATACCGATAGTCCAACCTA
SSR-1t4f: GACCTGACGACATAGACTTGAGAGAGCGACAAACGAAATGACCCCCAGCGATTATTCATTAC
SSR-1t18f: GACCTGACGACATAGACTTGAGAGAGCGACGCTTCTGGTCAGGCTGCGCAACTGTGTTATCC
SSR-1t2e: GACCTGACGACATAGACTTGAGAGAGCGACCTTAAACATCAGCTTGCTTTCGAGAAACAGTT
SSR-1t4e: GACCTGACGACATAGACTTGAGAGAGCGACCTCATCTTGAGGCAAAAGAATACACTCCCTCA
SSR-1t6e: GACCTGACGACATAGACTTGAGAGAGCGACGAATAAGGACGTAACAAAGCTGCTGACGGAAA
SSR-1t8e: GACCTGACGACATAGACTTGAGAGAGCGACCATAACCCGAGGCATAGTAAGAGCTTTTTAAG
SSR-1t10e: GACCTGACGACATAGACTTGAGAGAGCGACTTTTAATTGCCCGAAAGACTTCAATTCCAGAG
SSR-1t12e: GACCTGACGACATAGACTTGAGAGAGCGACTTTCATTTGGTCAATAACCTGTTTAATCAATA
SSR-1t14e: GACCTGACGACATAGACTTGAGAGAGCGACAGACAGTCATTCAAAAGGGTGAGATATCATAT
SSR-1t16e: GACCTGACGACATAGACTTGAGAGAGCGACGCTCATTTTCGCATTAAATTTTTGAGCTTAGA
SSR-1t18e: GACCTGACGACATAGACTTGAGAGAGCGACTTCGCCATTGCCGGAAACCAGGCAAACAGTAC
SSR1t4f: GACCTGACGACATAGACTTGAGAGAGCGACGAGCCGCCCCACCACCGGAACCGCCTAAAACA
SSR1t6f: GACCTGACGACATAGACTTGAGAGAGCGACTTATTCATAGGGAAGGTAAATATTCATTCAGT
SSR1t8f: GACCTGACGACATAGACTTGAGAGAGCGACAAAAGTAATATCTTACCGAAGCCCAACACTAT
SSR1t10f: GACCTGACGACATAGACTTGAGAGAGCGACCCTAATTTACGCTAACGAGCGTCTATATCGCG
SSR1t12f: GACCTGACGACATAGACTTGAGAGAGCGACATCGGCTGCGAGCATGTAGAAACCAGCTATAT
SSR1t14f: GACCTGACGACATAGACTTGAGAGAGCGACGCGTTATAGAAAAAGCCTGTTTAGAAGGCCGG
SSR1t16f: GACCTGACGACATAGACTTGAGAGAGCGACTTAAGACGTTGAAAACATAGCGATTTAAATCA
SSR1t18f: GACCTGACGACATAGACTTGAGAGAGCGACCTTTTACACAGATGAATATACAGTAAGCGCCA
SSR1t2e: GACCTGACGACATAGACTTGAGAGAGCGACTGCCTTGACTGCCTATTTCGGAACAGGGATAG
SSR1t4e: GACCTGACGACATAGACTTGAGAGAGCGACAACCAGAGACCCTCAGAACCGCCAGGGGTCAG
SSR1t16e: GACCTGACGACATAGACTTGAGAGAGCGACTAGAATCCCTGAGAAGAGTCAATAGGAATCAT
SSR1t18e: GACCTGACGACATAGACTTGAGAGAGCGACTTTAACGTTCGGGAGAAACAATAATTTTCCCT
SSR3t4f: GACCTGACGACATAGACTTGAGAGAGCGACGCCACCACTCTTTTCATAATCAAACCGTCACC
SSR3t18f: GACCTGACGACATAGACTTGAGAGAGCGACCCTGATTGAAAGAAATTGCGTAGACCCGAACG
SSR3t2e: GACCTGACGACATAGACTTGAGAGAGCGACAGTGTACTTGAAAGTATTAAGAGGCCGCCACC
SSR3t4e: GACCTGACGACATAGACTTGAGAGAGCGACGTTTGCCACCTCAGAGCCGCCACCGATACAGG
SSR3t16e: GACCTGACGACATAGACTTGAGAGAGCGACCTGTAAATCATAGGTCTGAGAGACGATAAATA

SSR3t18e: GACCTGACGACATAGACTTGAGAGAGCGACACAGAAATCTTTGAATACCAAGTTCCTTGCTT
SSR5t4f: GACCTGACGACATAGACTTGAGAGAGCGACCACCAGAGTTCGGTCATAGCCCCCGCCAGCAA
SSR5t18f: GACCTGACGACATAGACTTGAGAGAGCGACGCGCAGAGATATCAAAATTATTTGACATTATC
SSR5t2e: GACCTGACGACATAGACTTGAGAGAGCGACTAAGCGTCGAAGGATTAGGATTAGTACCGCCA
SSR5t4e: GACCTGACGACATAGACTTGAGAGAGCGACTCGGCATTCCGCCGCCAGCATTGACGTTCCAG
SSR5t16e: GACCTGACGACATAGACTTGAGAGAGCGACAAATCAATGGCTTAGGTTGGGTTACTAAATTT
SSR5t18e: GACCTGACGACATAGACTTGAGAGAGCGACAACCTACCGCGAATTATTCATTTCCAGTACAT
SSR7t4f: GACCTGACGACATAGACTTGAGAGAGCGACTGAGGCAGGCGTCAGACTGTAGCGTAGCAAGG
SSR7t6f: GACCTGACGACATAGACTTGAGAGAGCGACCCGGAAACACACCACGGAATAAGTAAGACTCC
SSR7t8f: GACCTGACGACATAGACTTGAGAGAGCGACTTATTACGGTCAGAGGGTAATTGAATAGCAGC
SSR7t10f: GACCTGACGACATAGACTTGAGAGAGCGACCTTTACAGTTAGCGAACCTCCCGACGTAGGAA
SSR7t12f: GACCTGACGACATAGACTTGAGAGAGCGACTCATTACCCGACAATAAACAACATATTTAGGC
SSR7t14f: GACCTGACGACATAGACTTGAGAGAGCGACAGAGGCATAATTTCATCTTCTGACTATAACTA
SSR7t16f: GACCTGACGACATAGACTTGAGAGAGCGACTATGTAAACCTTTTTAATGGAAAAATTACCT
SSR7t18f: GACCTGACGACATAGACTTGAGAGAGCGACGAGCAAAAACTTCTGAATAATGGAAGAAGGAG
SSR7t4e: GACCTGACGACATAGACTTGAGAGAGCGACTGCCTTTAGTCAGACGATTGGCCTGCCAGAAT
SSR7t6e: GACCTGACGACATAGACTTGAGAGAGCGACACGCAAAGGTCACCAATGAAACCAATCAAGTT
SSR7t8e: GACCTGACGACATAGACTTGAGAGAGCGACTGAACAAACAGTATGTTAGCAAACTAAAAGAA
SSR7t10e: GACCTGACGACATAGACTTGAGAGAGCGACGAGGCGTTAGAGAATAACATAAAAGAACACCC
SSR7t12e: GACCTGACGACATAGACTTGAGAGAGCGACCCAGACGAGCGCCCAATAGCAAGCAAGAACGC
SSR7t14e: GACCTGACGACATAGACTTGAGAGAGCGACTTTTAGTTTTTCGAGCCAGTAATAAATTCTGT
SSR7t16e: GACCTGACGACATAGACTTGAGAGAGCGACTTGAATTATGCTGATGCAAATCCACAAATATA
Other sequences can be found in the sequences list for the rectangle origami[4].

For the *eight-dot* pattern:

EDR-7t4e: AGAGTCTGACATGCTGCTCCATGAGAGGCTTTGAGGACTAGGGAGTT
EDR-7t6e: AGAGTCTGACATGCTCGATTTTAGAGGACAGATGAACGGCGCGACCT
EDR-5t6f: AGAGTCTGACATGCTCCAGGCGCTTAATCATTGTGAATTACAGGTAG
EDR-7t16e: AGAGTCTGACATGCTACCCGTCGTCATATGTACCCCGGTAAAGGCTA
EDR-7t18e: AGAGTCTGACATGCTATTAAGTTCGCATCGTAACCGTGCGAGTAACA
EDR-5t18f: AGAGTCTGACATGCTGTTTGAGGGAAAGGGGGATGTGCTAGAGGATC
EDR-1t4e: AGAGTCTGACATGCTCTCATCTTGAGGCAAAAGAATACACTCCCTCA
EDR1t4f: AGAGTCTGACATGCTGAGCCGCCCCACCACCGGAACCGCCTAAAACA
EDR-1t12e: AGAGTCTGACATGCTTTTCATTTGGTCAATAACCTGTTTAATCAATA
EDR1t12f: AGAGTCTGACATGCTATCGGCTGCGAGCATGTAGAAACCAGCTATAT
EDR-1t20e: AGAGTCTGACATGCTGCATAAAGTTCCACACAACATACGAAACAATT
EDR1t20f: AGAGTCTGACATGCTCGACAACTAAGTATTAGACTTTACAGCCGGAA
EDR7t4f: AGAGTCTGACATGCTTGAGGCAGGCGTCAGACTGTAGCGTAGCAAGG
EDR7t12f: AGAGTCTGACATGCTTCATTACCCGACAATAAACAACATATTTAGGC
EDR7t20f: AGAGTCTGACATGCTCGGAATTATTGAAAGGAATTGAGGTGAAAAAT
Other sequences can be found in the sequences list for the rectangle origami[4].

For *seven-dot* pattern:

SDR-7t10e: AGACTCTAACCTGCAGTCACAACAGTACGCTTTTTGCGCAGAAAACGAGAATGAATGTTTAG
SDR-7t12e: AGACTCTAACCTGCAGTCACAACAGTACGCCAAAATTAAAGTACGGTGTCTGGAAGAGGTCA
SDR-5t12f: AGACTCTAACCTGCAGTCACAACAGTACGCTCCATATACATACAGGCAAGGCAACTTTATTT
SDR-3t2f: AGACTCTAACCTGCAGTCACAACAGTACGCAAAAAAGGACAACCATCGCCCACGCGGGTAAA
SDR-3t4f: AGACTCTAACCTGCAGTCACAACAGTACGCATACGTAAAAGTACAACGGAGATTTCATCAAG
SDR-3t2e: AGACTCTAACCTGCAGTCACAACAGTACGCCAATGACACTCCAAAAGGAGCCTTACAACGCC
SDR-3t20f: AGACTCTAACCTGCAGTCACAACAGTACGCTCATAGCTACTCACATTAATTGCGCCCTGAGA
SDR-3t22f: AGACTCTAACCTGCAGTCACAACAGTACGCGAGTTGCACGAGATAGGGTTGAGTAAGGGAGC
SDR-3t20e: AGACTCTAACCTGCAGTCACAACAGTACGCGTGAGCTAGTTTCCTGTGTGAAATTTGGGAAG
SDR-1t10e: AGACTCTAACCTGCAGTCACAACAGTACGCTTTTAATTGCCCGAAAGACTTCAATTCCAGAG
SDR-1t12e: AGACTCTAACCTGCAGTCACAACAGTACGCTTTCATTTGGTCAATAACCTGTTTAATCAATA
SDR1t12f: AGACTCTAACCTGCAGTCACAACAGTACGCATCGGCTGCGAGCATGTAGAAACCAGCTATAT
SDR3t2f: AGACTCTAACCTGCAGTCACAACAGTACGCCTGAAACAGGTAATAAGTTTTAACCCCTCAGA
SDR3t4f: AGACTCTAACCTGCAGTCACAACAGTACGCGCCACCACTCTTTTCATAATCAAACCGTCACC

SDR3t2e: AGACTCTAACCTGCAGTCACAACAGTACGCAGTGTACTTGAAAGTATTAAGAGGCCGCCACC
SDR3t20f: AGACTCTAACCTGCAGTCACAACAGTACGCTTATTAATGCCGTCAATAGATAATCAGAGGTG
SDR3t22f: AGACTCTAACCTGCAGTCACAACAGTACGCAGGCGGTCATTAGTCTTTAATGCGCAATATTA
SDR3t20e: AGACTCTAACCTGCAGTCACAACAGTACGCAGATTAGATTTAAAAGTTTGAGTACACGTAAA
SDR5t10e: AGACTCTAACCTGCAGTCACAACAGTACGCAGGTTTTGAACGTCAAAAATGAAAGCGCTAAT
SDR5t12e: AGACTCTAACCTGCAGTCACAACAGTACGCAATGCAGACCGTTTTTATTTTCATCTTGCGGG
SDR7t12f: AGACTCTAACCTGCAGTCACAACAGTACGCTCATTACCCGACAATAAACAACATATTTAGGC
Other sequences can be found in the sequences list for the rectangle origami[4].

For the *rectangular block* pattern:

The following sequences in the tall rectangular origami were removed:
t1r12e, t1r14e, t1r16e, t1r18e, t1r20e, t1r12f, t1r14f, t1r16f, t1r18f, t1r20f, t-1r12e, t-1r14e, t-1r16e, t-1r18e, t-1r20e, t-1r12f, t-1r14f, t-1r16f, t-1r18f, t-1r20f.
Other sequences can be found in the sequences list for the tall rectangular origami[4].

For the *three-dot* pattern:

The following sequences in the triangular origami were removed:
A01, A05, A09, A13, A17, A33, A38, A42, A46, A50, B01, B05, B09, B13, B17, B33, B38, B42, B46, B50, C01, C05, C09, C13, C17, C33, C38, C42, C46, C50.
Other sequences can be found in the sequences list for the triangular origami[4].

For the small *digit 8* pattern:

mEt5r28e: GACCTGACGACATAGACTTGAGAGAGCGACGAAATGGAAAACATCGCCATTAAACAGAGGTG
mEt5r26e: GACCTGACGACATAGACTTGAGAGAGCGACAGGCGGTCTCTTTAGGAGCACTAAACATTTGA
mEt5r24e: GACCTGACGACATAGACTTGAGAGAGCGACGGATTTAGTTCATCAATATAATCCAGGGTTAG
mEt5r22e: GACCTGACGACATAGACTTGAGAGAGCGACAACCTACCGCGAATTATTCATTTCACATCAAG
mEt5r20e: GACCTGACGACATAGACTTGAGAGAGCGACAAAACAAACTGAGAAGAGTCAATATACCTTTT
mEt5r28f: GACCTGACGACATAGACTTGAGAGAGCGACTAGCCCTATTATTTACATTGGCAGCAATATTA
mEt5r26f: GACCTGACGACATAGACTTGAGAGAGCGACCTAAAATAAGTATTAACACCGCCTCGAACTGA
mEt5r24f: GACCTGACGACATAGACTTGAGAGAGCGACGATGGCAAAAGTATTAGACTTTACAAGGTTAT
mEt5r22f: GACCTGACGACATAGACTTGAGAGAGCGACGCGCAGAGATATCAAAATTATTTGTATCAGAT
mEt5r20f: GACCTGACGACATAGACTTGAGAGAGCGACTTAAGACGATTAATTACATTTAACACAAAATC
mEt3r28e: GACCTGACGACATAGACTTGAGAGAGCGACGTCACACGATTAGTCTTTAATGCGGCAACAGT
mEt3r20e: GACCTGACGACATAGACTTGAGAGAGCGACTTGAATTATTGAAAACATAGCGATTATAACTA
mEt3r28f: GACCTGACGACATAGACTTGAGAGAGCGACGAATGGCTACCAGTAATAAAAGGGCAAACTAT
mEt3r20f: GACCTGACGACATAGACTTGAGAGAGCGACTAGAATCCCCTTTTTAATGGAAACGGATTCG
mEt1r28e: GACCTGACGACATAGACTTGAGAGAGCGACGCCAACAGATACGTGGCACAGACATGAAAAAT
mEt1r20e: GACCTGACGACATAGACTTGAGAGAGCGACAAATCAATCGTCGCTATTAATTAAATCGCAAG
mEt1r28f: GACCTGACGACATAGACTTGAGAGAGCGACGCGTAAGAAGATAGAACCCTTCTGAACGCGCG
mEt1r26f: GACCTGACGACATAGACTTGAGAGAGCGACAAACCCTCTCACCTTGCTGAACCTAGAGGATC
mEt1r24f: GACCTGACGACATAGACTTGAGAGAGCGACATTTTGCGTTTAAAAGTTTGAGTACCGGCACC
mEt1r22f: GACCTGACGACATAGACTTGAGAGAGCGACCTTTTACACAGATGAATATACAGTGCCATCAA
mEt1r20f: GACCTGACGACATAGACTTGAGAGAGCGACCTGTAAATATATGTGAGTGAATAAAAAGGCTA
mEt-1r28e: GACCTGACGACATAGACTTGAGAGAGCGACGGGAGAGGCATTAATGAATCGGCCACCTGAAA
mEt-1r26e: GACCTGACGACATAGACTTGAGAGAGCGACCCCGGGTACCTGCAGGTCGACTCTCAAATATC
mEt-1r24e: GACCTGACGACATAGACTTGAGAGAGCGACGCTTCTGGCACTCCAGCCAGCTTTACATTATC
mEt-1r22e: GACCTGACGACATAGACTTGAGAGAGCGACAAATAATTTTTAACCAATAGGAACAACAGTAC
mEt-1r20e: GACCTGACGACATAGACTTGAGAGAGCGACTCAGGTCATTTTTGAGAGATCTACCCTTGCTT
mEt-1r28f: GACCTGACGACATAGACTTGAGAGAGCGACGCCAGCTGCGGTTTGCGTATTGGGAATCAAAA
mEt-1r20f: GACCTGACGACATAGACTTGAGAGAGCGACGGTAGCTATTGCCTGAGAGTCTGGTTAAATCA
mEt-3r28e: GACCTGACGACATAGACTTGAGAGAGCGACTGGTTTTTCTTTCCAGTCGGGAAAAATCATGG
mEt-3r20e: GACCTGACGACATAGACTTGAGAGAGCGACAGAGAATCAGCTGATAAATTAATGCTTTATTT
mEt-3r28f: GACCTGACGACATAGACTTGAGAGAGCGACACTGCCCGCTTTTCACCAGTGAGATGGTGGTT
mEt-3r20f: GACCTGACGACATAGACTTGAGAGAGCGACACCGTTCTGATGAACGGTAATCGTAATATTTT
mEt-5r28e: GACCTGACGACATAGACTTGAGAGAGCGACAGCTGATTACTCACATTAATTGCGTGTTATCC
mEt-5r26e: GACCTGACGACATAGACTTGAGAGAGCGACGCTCACAAGGGTAACGCCAGGGTTTTGGGAAG
mEt-5r24e: GACCTGACGACATAGACTTGAGAGAGCGACGGCGATCGCGCATCGTAACCGTGCGAGTAACA

mEt-5r22e: GACCTGACGACATAGACTTGAGAGAGCGACACCCGTCGTTAAATTGTAAACGTTAAAACTAG
mEt-5r20e: GACCTGACGACATAGACTTGAGAGAGCGACCATGTCAAAAATCACCATCAATATAACCCTCA
mEt-5r28f: GACCTGACGACATAGACTTGAGAGAGCGACGTGAGCTAGCCCTTCACCGCCTGGGGTTTGCC
mEt-5r26f: GACCTGACGACATAGACTTGAGAGAGCGACATTAAGTTTTCCACACAACATACGCCTAATGA
mEt-5r24f: GACCTGACGACATAGACTTGAGAGAGCGACTAGATGGGGTGCGGGCCTCTTCGCGCAAGGCG
mEt-5r22f: GACCTGACGACATAGACTTGAGAGAGCGACGCAAATATGATTCTCCGTGGGAACCGTTGGTG
mEt-5r20f: GACCTGACGACATAGACTTGAGAGAGCGACAGACAGTCTCATATGTACCCCGGTTTGTATAA
Other sequences can be found in the sequences list for the tall rectangle origami[4].

For the triangle origami patterns:

The sequence of ‘AGACTAGACTAGACT’ was used and linked to the 5 terminals of all the selected staple strands for the triangle DNA origami as shown in Fig. S28.

For the *C*，*u* patterns:

St5r6f: CTACCTCCAGCACCGTAGGGAAGGTAAATATTTTATTTTG
St5r8f: CTACCTCCTCACAATCCCGAGGAAACGCAATAATGAAATA
St5r10f: CTACCTCCGCAATAGCAGAGAATAACATAAAAACAGCCAT
St5r12f: CTACCTCCATTATTTATTAGCGAACCTCCCGACGTAGGAA
St5r14f: CTACCTCCTCATTACCGAACAAGAAAAATAATAATTCTGT
St5r16f: CTACCTCCCCAGACGACAAATTCTTACCAGTAGATAAATA
St5r18f: CTACCTCCAGGCGTTAGGCTTAGGTTGGGTTAAGCTTAGA
St5r20f: CTACCTCCTTAAGACGATTAATTACATTTAACACAAAATC
St5r22f: CTACCTCCGCGCAGAGATATCAAAATTATTTGTATCAGAT
St5r24f: CTACCTCCGATGGCAAAAGTATTAGACTTTACAAGGTTAT
St5r26f: CTACCTCCCTAAAATAAGTATTAACACCGCCTCGAACTGA
St3r4e: CTACCTCCGTTTGCCACCTCAGAGCCGCCACCGCCAGAAT
St3r6e: CTACCTCCTTATTCATGTCACCAATGAAACCATTATTAGC
St3r8e: CTACCTCCATACCCAAACACCACGGAATAAGTGACGGAAA
St3r10e: CTACCTCCGCGCATTAATAAGAGCAAGAAACAATAACGGA
St3r12e: CTACCTCCAGGTTTTGGCCAGTTACAAAATAAACAGGGAA
St3r14e: CTACCTCCCTAATTTACCGTTTTTATTTTCATCTTGCGGG
St3r16e: CTACCTCCACGCTCAACGACAAAAGGTAAAGTATCCCATC
St3r18e: CTACCTCCTATGTAAAGAAATACCGACCGTGTTAAAGCCA
St3r20e: CTACCTCCTTGAATTATTGAAAACATAGCGATTATAACTA
St3r22e: CTACCTCCACAGAAATCTTTGAATACCAAGTTAATTTCAT
St3r24e: CTACCTCCCGACAACTTCATCATATTCCTGATCACGTAAA
St3r26e: CTACCTCCGCCACGCTTTGAAAGGAATTGAGGAAACAATT
St3r6f: CTACCTCCCCGGAAACTAAAGGTGAATTATCATAAAAGAA
St3r26f: CTACCTCCATCAACAGGAGAGCCAGCAGCAAAATATTTTT
St1r4e: CTACCTCCAACCAGAGACCCTCAGAACCGCCACGTTCCAG
St1r6e: CTACCTCCGACTTGAGGTAGCACCATTACCATATCACCGG
St1r24e: CTACCTCCTTATTAATGAACAAAGAAACCACCTTTTCAGG
St1r26e: CTACCTCCCTAAAGCAAATCAATATCTGGTCACCCGAACG
St1r6f: CTACCTCCAATCACCACCATTTGGGAATTAGACCAACCTA
St1r26f: CTACCTCCAAACCCTCTCACCTTGCTGAACCTAGAGGATC
St-1r4e: CTACCTCCCAATGACAGCTTGATACCGATAGTCTCCCTCA
St-1r6e: CTACCTCCAAACGAAATGCCACTACGAAGGCAGCCAGCAA
St-1r24e: CTACCTCCGCTTCTGGCACTCCAGCCAGCTTTACATTATC
St-1r26e: CTACCTCCCCCGGGTACCTGCAGGTCGACTCTCAAATATC
St-1r6f: CTACCTCCATACGTAAGAGGCAAAAGAATACACTGACCAA
St-1r26f: CTACCTCCCTTGCATGCCGAGCTCGAATTCGTCCTGTCGT
St-3r4e: CTACCTCCATATATTCTCAGCTTGCTTTCGAGTGGGATTT
St-3r6e: CTACCTCCCTCATCTTGGAAGTTTCCATTAAACATAACCG
St-3r24e: CTACCTCCTTCGCCATGGACGACGACAGTATCGTAGCCAG
St-3r26e: CTACCTCCTCATAGCTTGTAAAACGACGGCCAAAGCGCCA
St-3r6f: CTACCTCCTTTCATGATGACCCCCAGCGATTAAGGCGCAG
St-3r8f: CTACCTCCACGGTCAATGACAAGAACCGGATATGGTTTAA

St-3r14f: CTACCTCCTCAGAAGCCTCCAACAGGTCAGGATTTAAATA
St-3r16f: CTACCTCCTGCAACTAGGTCAATAACCTGTTTAGAATTAG
St-3r18f: CTACCTCCCAAAATTAGGATAAAAATTTTTAGGATATTCA
St-3r20f: CTACCTCCACCGTTCTGATGAACGGTAATCGTAATATTTT
St-3r22f: CTACCTCCGTTAAAATAACATTAAATGTGAGCATCTGCCA
St-3r24f: CTACCTCCGTTTGAGGTCAGGCTGCGCAACTGTTCCCAGT
St-3r26f: CTACCTCCCACGACGTGTTTCCTGTGTGAAATTTGCGCTC
St-5r6e: CTACCTCCGCGAAACAAGAGGCTTTGAGGACTAGGGAGTT
St-5r8e: CTACCTCCCCAAATCATTACTTAGCCGGAACGTACCAAGC
St-5r14e: CTACCTCCTACCTTTAAGGTCTTTACCCTGACAATCGTCA
St-5r16e: CTACCTCCTTTCATTTCTGTAGCTCAACATGTTTAGAGAG
St-5r18e: CTACCTCCTATATTTTCATACAGGCAAGGCAAAGCTATAT
St-5r20e: CTACCTCCCATGTCAAAAATCACCATCAATATAACCCTCA
St-5r22e: CTACCTCCACCCGTCGTTAAATTGTAAACGTTAAAACTAG
St-5r24e: CTACCTCCGGCGATCGCGCATCGTAACCGTGCGAGTAACA
St-5r26e: CTACCTCCGCTCACAAGGGTAACGCCAGGGTTTTGGGAAG
Other sequences can be found in the sequences list for the tall rectangle origami[4].

SCu GGAGGTAGGACAAGCACGATACCATGCGTCTTCGGTAG

For the *A*, *g* patterns:

Et5r6f: CTTGGGTCGTAGACATCGACGACTACTGACAGCACCGTAGGGAAGGTAAATATTTTATTTTG
Et5r8f: CTTGGGTCGTAGACATCGACGACTACTGACTCACAATCCCGAGGAAACGCAATAATGAAATA
Et5r10f:CTTGGGTCGTAGACATCGACGACTACTGACGCAATAGCAGAGAATAACATAAAAACAGCCAT
Et5r12f: CTTGGGTCGTAGACATCGACGACTACTGACATTATTTATTAGCGAACCTCCCGACGTAGGAA
Et5r14f: CTTGGGTCGTAGACATCGACGACTACTGACTCATTACCGAACAAGAAAAATAATAATTCTGT
Et5r16f: CTTGGGTCGTAGACATCGACGACTACTGACCCAGACGACAAATTCTTACCAGTAGATAAATA
Et5r18f: CTTGGGTCGTAGACATCGACGACTACTGACAGGCGTTAGGCTTAGGTTGGGTTAAGCTTAGA
Et5r20f: CTTGGGTCGTAGACATCGACGACTACTGACTTAAGACGATTAATTACATTTAACACAAAATC
Et5r22f: CTTGGGTCGTAGACATCGACGACTACTGACGCGCAGAGATATCAAAATTATTTGTATCAGAT
Et5r24f: CTTGGGTCGTAGACATCGACGACTACTGACGATGGCAAAAGTATTAGACTTTACAAGGTTAT
Et5r26f: CTTGGGTCGTAGACATCGACGACTACTGACCTAAAATAAGTATTAACACCGCCTCGAACTGA
Et3r4e: CTTGGGTCGTAGACATCGACGACTACTGACGTTTGCCACCTCAGAGCCGCCACCGCCAGAAT
Et3r6e: CTTGGGTCGTAGACATCGACGACTACTGACTTATTCATGTCACCAATGAAACCATTATTAGC
Et3r8e: CTTGGGTCGTAGACATCGACGACTACTGACATACCCAAACACCACGGAATAAGTGACGGAAA
Et3r10e: CTTGGGTCGTAGACATCGACGACTACTGACGCGCATTAATAAGAGCAAGAAACAATAACGGA
Et3r12e: CTTGGGTCGTAGACATCGACGACTACTGACAGGTTTTGGCCAGTTACAAAATAAACAGGGAA
Et3r14e: CTTGGGTCGTAGACATCGACGACTACTGACCTAATTTACCGTTTTTATTTTCATCTTGCGGG
Et3r16e: CTTGGGTCGTAGACATCGACGACTACTGACACGCTCAACGACAAAAGGTAAAGTATCCCATC
Et3r18e: CTTGGGTCGTAGACATCGACGACTACTGACTATGTAAAGAAATACCGACCGTGTTAAAGCCA
Et3r20e: CTTGGGTCGTAGACATCGACGACTACTGACTTGAATTATTGAAAACATAGCGATTATAACTA
Et3r22e: CTTGGGTCGTAGACATCGACGACTACTGACACAGAAATCTTTGAATACCAAGTTAATTTCAT
Et3r24e: CTTGGGTCGTAGACATCGACGACTACTGACCGACAACTTCATCATATTCCTGATCACGTAAA
Et3r26e: CTTGGGTCGTAGACATCGACGACTACTGACGCCACGCTTTGAAAGGAATTGAGGAAACAATT
Et3r6f: CTTGGGTCGTAGACATCGACGACTACTGACCCGGAAACTAAAGGTGAATTATCATAAAAGAA
Et3r16f: CTTGGGTCGTAGACATCGACGACTACTGACTAAAGTACCAGTAGGGCTTAATTGCTAAATTT
Et3r26f: CTTGGGTCGTAGACATCGACGACTACTGACATCAACAGGAGAGCCAGCAGCAAAATATTTTT
Et1r4e: CTTGGGTCGTAGACATCGACGACTACTGACAACCAGAGACCCTCAGAACCGCCACGTTCCAG
Et1r6e: CTTGGGTCGTAGACATCGACGACTACTGACGACTTGAGGTAGCACCATTACCATATCACCGG
Et1r14e: CTTGGGTCGTAGACATCGACGACTACTGACATCGGCTGACCAAGTACCGCACTCTTAGTTGC
Et1r16e: CTTGGGTCGTAGACATCGACGACTACTGACCATATTTATTTCGAGCCAGTAATAAATCAATA
Et1r24e: CTTGGGTCGTAGACATCGACGACTACTGACTTATTAATGAACAAAGAAACCACCTTTTCAGG
Et1r26e: CTTGGGTCGTAGACATCGACGACTACTGACCTAAAGCAAATCAATATCTGGTCACCCGAACG
Et1r6f: CTTGGGTCGTAGACATCGACGACTACTGACAATCACCACCATTTGGGAATTAGACCAACCTA
Et1r16f: CTTGGGTCGTAGACATCGACGACTACTGACAGAGGCATACAACGCCAACATGTATCTGCGAA
Et1r26f: CTTGGGTCGTAGACATCGACGACTACTGACAAACCCTCTCACCTTGCTGAACCTAGAGGATC
Et-1r4e: CTTGGGTCGTAGACATCGACGACTACTGACCAATGACAGCTTGATACCGATAGTCTCCCTCA

Et-1r6e: CTTGGGTCGTAGACATCGACGACTACTGACAAACGAAATGCCACTACGAAGGCAGCCAGCAA
Et-1r14e: CTTGGGTCGTAGACATCGACGACTACTGACTTTTAATTGCCCGAAAGACTTCAACAAGAACG
Et-1r16e: CTTGGGTCGTAGACATCGACGACTACTGACCGAGTAGAACAGTTGATTCCCAATATTTAGGC
Et-1r24e: CTTGGGTCGTAGACATCGACGACTACTGACGCTTCTGGCACTCCAGCCAGCTTTACATTATC
Et-1r26e: CTTGGGTCGTAGACATCGACGACTACTGACCCCGGGTACCTGCAGGTCGACTCTCAAATATC
Et-1r6f: CTTGGGTCGTAGACATCGACGACTACTGACATACGTAAGAGGCAAAAGAATACACTGACCAA
Et-1r16f: CTTGGGTCGTAGACATCGACGACTACTGACTCCATATATTTAGTTTGACCATTAAGCATAAA
Et-1r26f: CTTGGGTCGTAGACATCGACGACTACTGACCTTGCATGCCGAGCTCGAATTCGTCCTGTCGT
Et-3r4e: CTTGGGTCGTAGACATCGACGACTACTGACATATATTCTCAGCTTGCTTTCGAGTGGGATTT
Et-3r6e: CTTGGGTCGTAGACATCGACGACTACTGACCTCATCTTGGAAGTTTCCATTAAACATAACCG
Et-3r14e: CTTGGGTCGTAGACATCGACGACTACTGACGAAGCAAAAAAGCGGATTGCATCAATGTTTAG
Et-3r16e: CTTGGGTCGTAGACATCGACGACTACTGACTCGCAAATAAGTACGGTGTCTGGACCAGACCG
Et-3r24e: CTTGGGTCGTAGACATCGACGACTACTGACTTCGCCATGGACGACGACAGTATCGTAGCCAG
Et-3r26e: CTTGGGTCGTAGACATCGACGACTACTGACTCATAGCTTGTAAAACGACGGCCAAAGCGCCA
Et-3r6f: CTTGGGTCGTAGACATCGACGACTACTGACTTTCATGATGACCCCCAGCGATTAAGGCGCAG
Et-3r8f: CTTGGGTCGTAGACATCGACGACTACTGACACGGTCAATGACAAGAACCGGATATGGTTTAA
Et-3r10f: CTTGGGTCGTAGACATCGACGACTACTGACTTTCAACTACGGAACAACATTATTAACACTAT
Et-3r12f: CTTGGGTCGTAGACATCGACGACTACTGACCATAACCCGCGTCCAATACTGCGGTATTATAG
Et-3r14f: CTTGGGTCGTAGACATCGACGACTACTGACTCAGAAGCCTCCAACAGGTCAGGATTTAAATA
Et-3r16f: CTTGGGTCGTAGACATCGACGACTACTGACTGCAACTAGGTCAATAACCTGTTTAGAATTAG
Et-3r18f: CTTGGGTCGTAGACATCGACGACTACTGACCAAAATTAGGATAAAAATTTTTAGGATATTCA
Et-3r20f: CTTGGGTCGTAGACATCGACGACTACTGACACCGTTCTGATGAACGGTAATCGTAATATTTT
Et-3r22f: CTTGGGTCGTAGACATCGACGACTACTGACGTTAAAATAACATTAAATGTGAGCATCTGCCA
Et-3r24f: CTTGGGTCGTAGACATCGACGACTACTGACGTTTGAGGTCAGGCTGCGCAACTGTTCCCAGT
Et-3r26f: CTTGGGTCGTAGACATCGACGACTACTGACCACGACGTGTTTCCTGTGTGAAATTTGCGCTC
Et-5r6e: CTTGGGTCGTAGACATCGACGACTACTGACGCGAAACAAGAGGCTTTGAGGACTAGGGAGTT
Et-5r8e: CTTGGGTCGTAGACATCGACGACTACTGACCCAAATCATTACTTAGCCGGAACGTACCAAGC
Et-5r10e: CTTGGGTCGTAGACATCGACGACTACTGACAAAGATTCTAAATTGGGCTTGAGATTCATTAC
Et-5r12e: CTTGGGTCGTAGACATCGACGACTACTGACTAAATATTGAGGCATAGTAAGAGCACAGGTAG
Et-5r14e: CTTGGGTCGTAGACATCGACGACTACTGACTACCTTTAAGGTCTTTACCCTGACAATCGTCA
Et-5r16e: CTTGGGTCGTAGACATCGACGACTACTGACTTTCATTTCTGTAGCTCAACATGTTTAGAGAG
Et-5r18e: CTTGGGTCGTAGACATCGACGACTACTGACTATATTTTCATACAGGCAAGGCAAAGCTATAT
Et-5r20e: CTTGGGTCGTAGACATCGACGACTACTGACCATGTCAAAAATCACCATCAATATAACCCTCA
Et-5r22e: CTTGGGTCGTAGACATCGACGACTACTGACACCCGTCGTTAAATTGTAAACGTTAAAACTAG
Et-5r24e: CTTGGGTCGTAGACATCGACGACTACTGACGGCGATCGCGCATCGTAACCGTGCGAGTAACA
Et-5r26e: CTTGGGTCGTAGACATCGACGACTACTGACGCTCACAAGGGTAACGCCAGGGTTTTGGGAAG
Other sequences can be found in the sequences list for the tall rectangle origami[4].

Staple for assembly of the origami of *C*, *u*, *A*, *g* patterns

L-7r2i: CTGAAACAATAGGAACCCATGTACAGGGATAGCAAGCCCA
L-7r6e: TGCCTTTAGACAGCATCGGAACGAACCCTCAG
L-7r10e: AAAAGTAACTTGCCCTGACGAGAACATTCAGT
L-7r14e: TATAGAAGCAGAAAACGAGAATGAAATGCTTT
L-7r18e: AATTACTAACTAATAGTAGTAGCAAGGTGGCA
L-7r22e: GAGCAAAACCCAAAAACAGGAAGATGATAATC
L-7r26e: AGATTAGAGAAAGGGGGATGTGCTTATTACGC
L-7r30e: GGAAATACGCAAGCGGTCCACGCTCCCTGAGA
L7r2f: AATAATAAGTCAGACGATTGGCCTCAGGAGGT
L7r6f: CGCCTGATAGACAAAAGGGCGACAGGTTTACC
L7r10f: CATTCAACAACGTCAAAAATGAAAAAACGATT
L7r14f: TTTTGCGACGCGCCTGTTTATCAGTTCAGCT
L7r18f: AGGTAAAG CATAGGTCTGAGAGACGTGAATTT
L7r22f: GATTGACCACTTCTGAATAATGGATGATTGTT
L7r26f: GCATAAAGCCAGCAGAAGATAAAAAATACCGA
L7r30j: ACCCAAATCAGGAACGGTACGCCATTAAAGGGATTTTAGA
L-7r4e: TGAGGCAGTTTTTTCACGTTGAAAGAATTGCG
L-7r8e: AGCGCCAAAAATTGTGTCGAAATCTGTATCAT

L-7r12e: TTTTGTTTTAATGCAGATACATAAGAATACCA
L-7r16e: AATGCAGAGATGGCTTAGAGCTTAAGAGGTCA
L-7r20e: ATCAAAATATTCAAAAGGGTGAGATAATGTGT
L-7r24e: TGGATTATGTAATGGGATAGGTCAAAACGGCG
L-7r28e: ACGAACCATGTAAAGCCTGGGGTGAGCCGGAA
L7r4f: CAGCGAAAGCGTCAGACTGTAGCGATCAAGTT
L7r8f: GAATAAGGGCAGATAGCCGAACAATTTTTAAG
L7r12f: AAACAGTTGCTTATCCGGTATTCTAAATCAGA
L7r16f: TCAATTCTGAAAAAGCCTGTTTAGGGAATCAT
L7r20f: AGAAAAGCGAAGATGATGAAACAAAATTACCT
L7r24f: CAGCTGGCGCCGTCAATAGATAATCAACTAAT
L7r28f: GAGTTGCACTACATTTTGACGCTCACGCTCAT

Supplementary References:

1. Biesinger, M. C., Lau, L.W.M., Gerson, A. R. & Smart, R.St, C. Resolving surface chemical states in XPS analysis of first row transition metals, oxides and hydroxides: Sc, Ti, V, Cu and Zn. *Appl. Surf. Sci.* **257**, 887-898 (2010).
2. Ferraria, A. M., Carapeto, A. P. & Botelho do Rego, A. M. B. X-ray photoelectron spectroscopy: Silver salts revisited. *Vacuum* **86**, 1988-1991 (2012).
3. Liang, X., Mochizuki, T., & Asanuma, H. A supra-photoswitch involving sandwiched DNA base pairs and azobenzenes for light-driven nanostructures and nanodevices. *Small* **5**, 1761-1768 (2009).
4. Rothemund, P. W. K. Folding DNA to create nanoscale shapes and patterns. *Nature* **440**, 297-302 (2006).
